# Advancing the Exploration of the Ubiquitin‐like Protein FUBI with Synthetic Chemical Tools

**DOI:** 10.1002/cbic.202500321

**Published:** 2025-06-23

**Authors:** Francesca D’Amico, Cami M. P. Talavera Ormeño, Shivanganie Poeran, Jimmy Akkermans, Rayman T. N. Tjokrodirijo, Bharath Sampadi, Peter van Veelen, Aysegul Sapmaz, Monique P. C. Mulder

**Affiliations:** ^1^ Department of Cell and Chemical Biology Leiden University Medical Center (LUMC) Einthovenweg 20 2333 ZC Leiden The Netherlands; ^2^ Center for Proteomics and Metabolomics Leiden University Medical Center (LUMC) Einthovenweg 20 2333 ZC Leiden The Netherlands

**Keywords:** activity‐based probes, chemical biology, FUBI, post‐translational modifications, Ub‐like proteins

## Abstract

The Ubiquitin‐like protein FUBI is encoded in humans by the FAU gene, whose down‐regulation in prostate, ovarian and breast cancer is significantly associated with poor prognosis. Despite its implications in disease progression, the regulatory mechanisms orchestrated by FUBI remain elusive. To address this knowledge gap, a linear synthetic platform is developed to generate FUBI chemical tools, enabling the site‐specific incorporation of unnatural building blocks and the introduction of fluorophores, tags, and reactive warheads. Using this platform, activity‐based probes are created for FUBI conjugation and deconjugation enzymes, validating them in cell lysate‐based assays and proteomics. Additionally, a triazole‐linked Di‐FUBI is synthesized to investigate FUBI chain modulators. Among the proteomics hits, IMPDH1 and the deubiquitinase UCHL3 are identified as novel Di‐FUBI specific interactors. Further characterization revealed that Di‐FUBI inhibits UCHL3 cleavage activity in a concentration‐dependent manner, suggesting a novel regulatory interplay between UCHL3 and FUBI. Collectively, these tools demonstrate the versatility of the synthetic FUBI platform, advancing the characterization of FUBI‐related enzymes in the ongoing efforts to decipher the complex code of ubiquitin‐like signaling.

## Graphical abstract

This study unveils a novel synthetic platform by solid‐phase peptide synthesis for generating FUBI‐based chemical tools to investigate FUBIylation, a poorly understood ubiquitin‐like signaling process linked to cancer. By employing these innovative probes, we uncover novel FUBI interactors, paving the way for transformative insights into FUBI biology and its therapeutic potential.

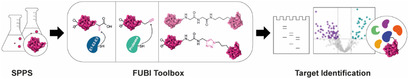

## 1 Introduction

Post‐translational modifications (PTMs) indicate the covalent and reversible attachment of chemical groups to substrate proteins, dynamically affecting their stability, localization, and activity.^[^ ^1^ ^]^ Ubiquitination, a highly conserved PTM in eukaryotic cells, canonically results in the formation of an isopeptide bond between the Lys side chain(s) of the substrate and the C‐terminal Gly of ubiquitin (Ub).^[^ ^2^ ^]^ This process is orchestrated by an enzymatic cascade involving E1‐activating enzyme, E2‐conjugating enzyme and E3 ligases, and it is reversed by deubiquitinases (DUBs).^[^ ^3^ ^]^ Ubiquitin‐like proteins (Ubls) constitute a diverse group of post‐translational modifiers, which are structurally and mechanistically related to Ub (Figure S1, Supporting Information), but exhibit distinct roles in cellular processes.^[^ ^4^ ^]^ Similar to Ub, Ubls are reversibly conjugated to substrates modulating their conformation, interactions, and functions.^[^ ^5^ ^]^ One of the least‐studied Ubl modifiers is FUBI, a protein consisting of 74 amino acids encoded by the FAU (Finkel Biskis‐Reilly‐Murine Sarcoma virus associated ubiquitously expressed) gene.^[^ ^6^ ^]^ FUBI plays a role in immunoregulation^[^ ^7^, ^8^, ^9^ ^]^ and has a pro‐apoptotic function in prostate, ovarian and breast cancers^[^ ^10^ ^]^ where its down‐regulation is strongly linked to poor prognosis.^[^ ^11^ ^]^ FUBI is expressed as an N‐terminal fusion with the ribosomal protein eS30,^[^ ^12^ ^]^ and the maturation of the 40S ribosomal subunit relies on FUBI's proteolytic cleavage by the nucleolar protease USP36.^[^ ^13^, ^14^ ^]^ Recent work has shown cross‐reactivity of USP16 toward Ub, ISG15, and FUBI.^[^ ^15^, ^16^ ^]^ Despite its potential as pharmacological target, the regulatory mechanisms linked to FUBI are still poorly understood. It is unclear whether other proteases can process FUBI, and although FUBIylation has been observed on substrates related to immunomodulation‐ and apoptosis^[^ ^8^, ^9^, ^10^, ^17^ ^]^ the topology of FUBI conjugation and the associated enzymes have yet to be investigated.

**Figure 1:**
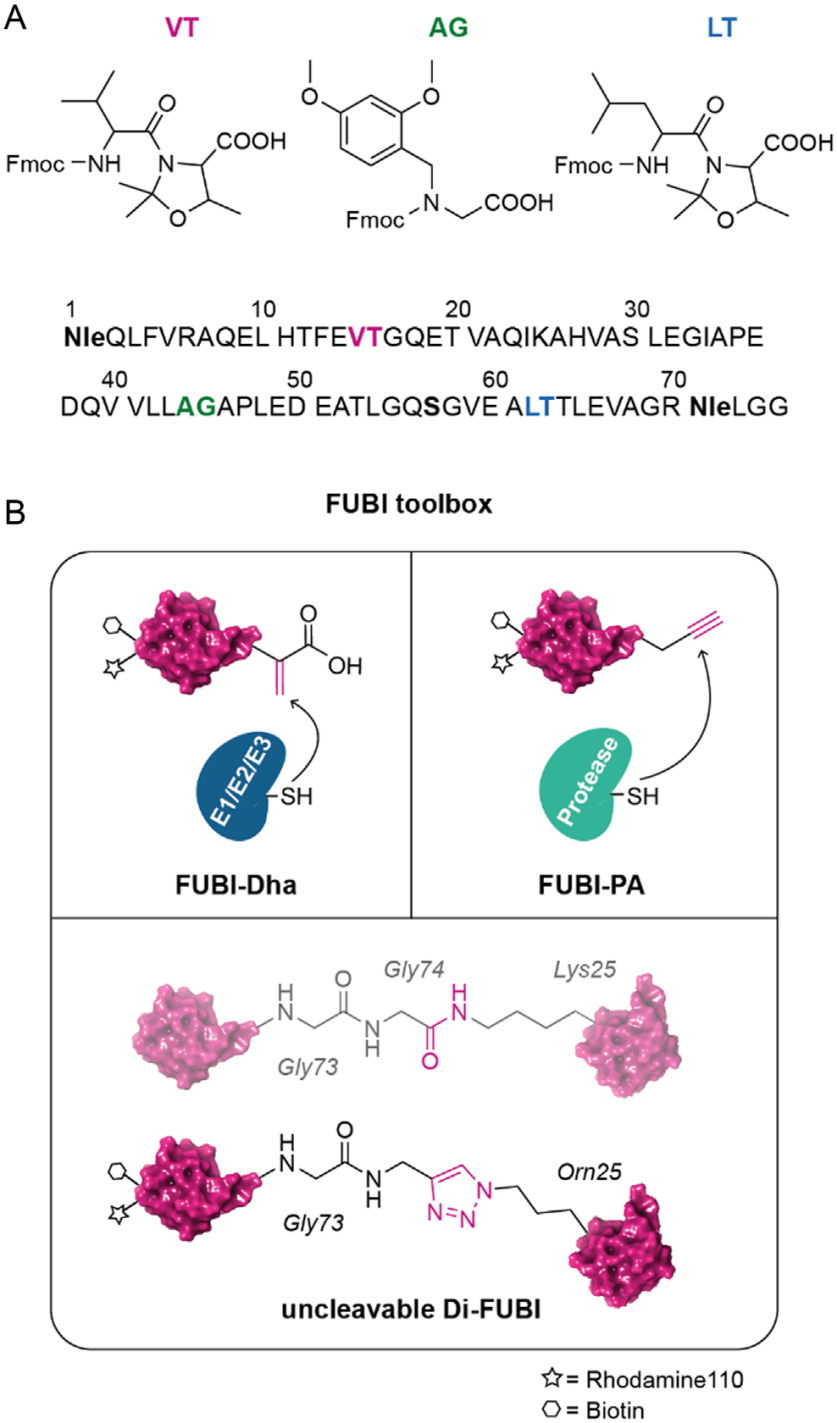
Generation of the FUBI toolbox. **A)** Synthetic sequence for FUBI SPPS. Strategic incorporation of VT, AG, and LT dipeptides prevents aggregation and facilitates peptide elongation. Cysteine is replaced with its isostere Serine to avoid disulfide bond formation, while Methionine is replaced with Norleucine (Nle) to prevent side chain oxidation. **B)** Schematic representation of the FUBI toolbox synthesized in this work. Rho/Biotin‐FUBI‐Dha and Rho/Biotin‐FUBI‐PA are designed to study conjugation (E1, E2, E3) and deconjugation (proteases) enzymes, respectively. Additionally, Rho/Biotin‐triazole‐linked‐Di‐FUBI serves as a stable, uncleavable mimic of native Di‐FUBI, enabling the investigation of FUBI chain interactors.

To systematically explore the components of the FUBI machinery, FUBI‐based peptides and probes can serve as powerful tools for biochemical characterization. Recently, ABPs for FUBI deconjugating enzymes have been obtained semi‐synthetically using intein chemistry.^[^ ^18^ ^]^ Building on the expanded repertoire of reagents and activity‐based probes (ABPs) developed for Ub conjugation and deconjugation enzymes,^[^ ^19^ ^]^ along with recent advancements in the Ubls field,^[^ ^20^ ^]^ we aimed to develop a versatile synthetic platform for FUBI (**Figure** **1**A, S2A, Supporting Information). In this work, we describe the generation of a FUBI toolbox via total linear solid‐phase peptide synthesis (SPPS), allowing the site‐selective incorporation of non‐natural amino acids and the introduction of fluorescent dyes, tags and reactive warheads through N‐ and C‐terminal labeling strategies. This approach enables diverse biochemical applications to identify specific components of the FUBI machinery (Figure 1B).

## 2 Results and Discussion

### 2.1 Chemical Synthesis of FUBI and ABPs

To enable the successful linear synthesis of FUBI full‐length, we strategically incorporated aggregation breakers (pseudoproline in VT and LT dipeptides and dimethoxybenzyl in AG dipeptide) to increase coupling efficiency during peptide elongation. Concurrently, isosteric single‐point mutations were introduced to improve product stability and purity, while maintaining the peptide's structural integrity: Cys was replaced with Ser to prevent disulfide bridging, and Met with Norleucine (Nle) to avoid oxidation of the sulfur‐containing side chain^[^ ^21^ ^]^ (Figure 1A, S2A, Supporting Information). With a working synthesis in hands, we prepared FUBI‐ABPs meant to covalently capture enzymes harboring an active site cysteine (Figure 1B).^[^ ^22^ ^]^ The design of our FUBI‐ABPs includes the FUBI sequence as a *recognition element*, with a Rhodamine (Rho) and Biotin‐containing residue at the N‐terminal serving as both a *fluorophore* and *reporter tag*, and an electrophilic moiety at the C‐terminal functions as Cys‐reactive warhead^[^ ^23^ ^]^ (Figure S2B, Supporting Information).

Covalent modification of the Cys enzymatic active site by the ABP allows for the isolation and characterization of captured proteins, aiding in the identification of new players involved in FUBI deconjugation and conjugation. Previous studies have shown that Ub‐Propargyl (Ub‐PA) can effectively trap proteases (DUBs),^[^ ^24^ ^]^ while Ub‐Dehydroalanine (Ub‐Dha) probes characterized conjugation enzymes (E1, E2, E3).^[^ ^25^, ^26^, ^27^ ^]^ In line with these observations, we synthesized the dual‐label probes Rho/Biotin‐FUBI‐PA (FUBI‐PA) and Rho/Biotin‐FUBI‐Dha (FUBI‐Dha) to covalently and specifically target FUBI deconjugation and conjugation enzymes, respectively (Figure 1B). This strategy enables both the visualization and affinity purification of captured enzymes. To achieve this, we prepared FUBI‐dG on a hyper‐acid‐labile Chlorotrytil resin using standard Fmoc‐based SPPS coupling conditions.^[^ ^28^ ^]^ Afterward, the N‐terminus was functionalized by coupling a PEG_2_ spacer and a NH_2_‐Lys(Biotin)‐OH residue, which was then modified with N,N’‐Boc‐protected 5‐carboxyrhodamine (Rhodamine 110). Following cleavage of the protected peptide from the resin with 20% hexafluoroisopropanol (HFIP) in DCM (Figure S2C, Supporting Information), the C‐terminus was further modified to introduce the warheads. Specifically, the FUBI‐PA probe was obtained by directly coupling propargylamine to the C‐terminus of the protected FUBI‐dG (Figure S2D, Supporting Information). Whereas, the Dha probe was synthesized by first coupling NH_2_‐Cys(Bz)‐OMe to the C‐terminus, followed by an oxidative elimination reaction with synthesized O‐esitylenesulfonylhydroxylamine (MSH)^[^ ^29^ ^]^ and hydrolysis of the resulting methyl ester^[^ ^30^ ^]^ (Figure S2D, Supporting Information). Global deprotection and HPLC purification generated FUBI‐PA and FUBI‐Dha in high purity (Figure S3, Supporting Information).

**Figure 2:**
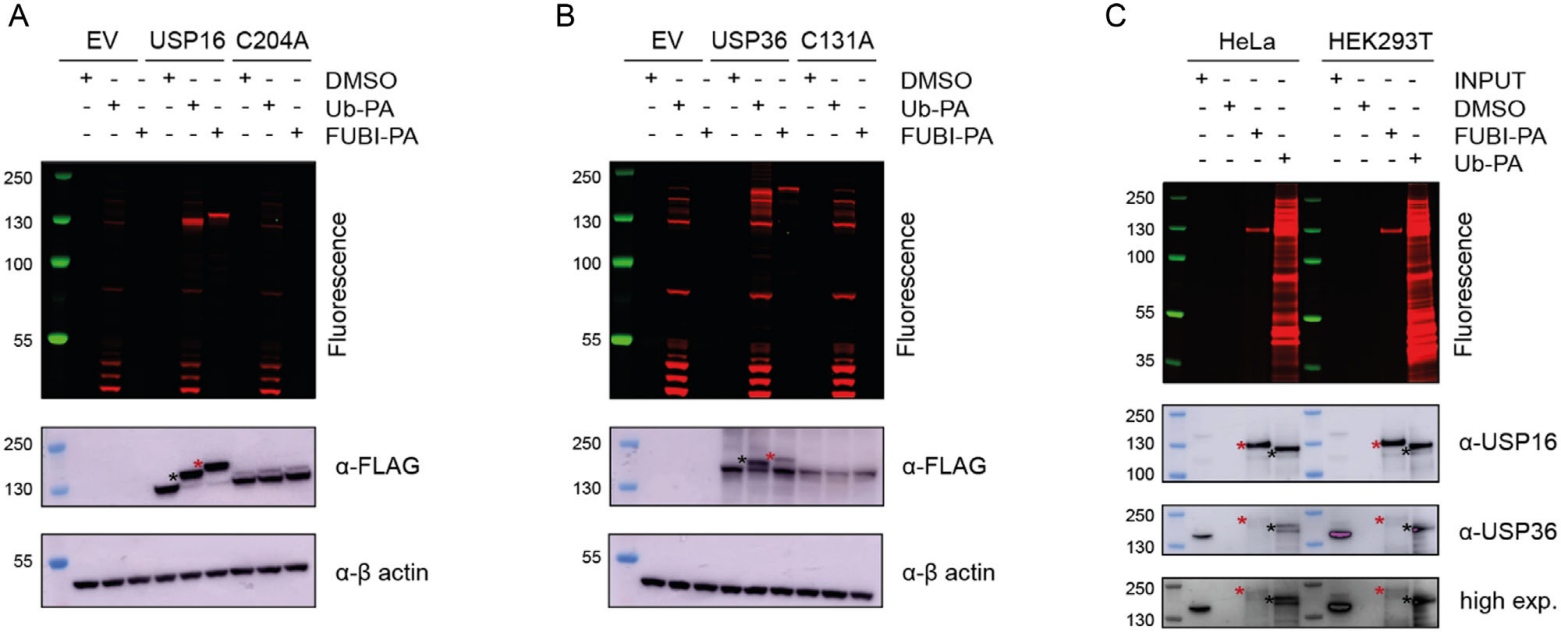
Validation of synthetic Rho/Biotin‐FUBI‐PA in cell lysate‐based assays. A) Labeling of ectopically expressed full‐length FLAG‐USP16 (WT and C204A catalytically dead mutant) and B) ectopically expressed Full‐length FLAG‐USP36 (WT and C131A catalytically dead mutant) in HEK293T cell lysates. C) Pulldown of endogenous USP16 and USP36 (EV = empty vector; INPUT = cell lysate prior to pulldown, without probe or control; red asterisks indicate protein:FUBI‐PA complexes; black asterisks indicate protein:Ub‐PA complexes).

To validate our synthetic probes, we evaluated FUBI‐PA against the previously identified FUBI‐reactive proteases USP16 and USP36.^[^ ^18^ ^]^ To this end, we ectopically expressed full‐length FLAG‐USP16 and FLAG‐USP36 in HEK293T cells (either WT or CA catalytic‐dead mutants) and performed cell lysate activity‐based labeling experiments. The successful detection of protein:ABP complex formation, which was absent in samples containing catalytically inactive USPs, demonstrated that the chemically synthesized probe maintained biologically relevant folding and activity (**Figure** **2**A,B). This result was further corroborated by the effective pulldown of endogenous USP16 and USP36 with synthetic FUBI‐PA either in HeLa or HEK293T cell lysates (Figure 2C). Building on these findings, we used FUBI‐PA to profile covalent interactors by proteomics analysis in HEK293T cells. This approach demonstrated significant enrichment of both USP16 and USP36 in samples treated with the covalent probe (**Figure** **3**A, S4, Supporting Information).

Figure 3Proteomics results from covalent pulldown with Rho/Biotin‐FUBI‐PA and Rho/Biotin‐FUBI‐Dha in HEK293T cell lysates. A) Volcano plot from FUBI‐PA pulldown highlights covalent and specific FUBI interactors after filtering non‐covalent interactors using FUBI‐FL (negative control, Figure S4, Supporting Information). B) Scatter plot of cross‐comparative analysis of FUBI‐Dha/DMSO (negative control 1, Figure S6A, Supporting Information) and FUBI‐Dha/FUBI‐Dha + Apyrase (negative control 2, Figure S6B, Supporting Information) to identify specific covalent and ATP‐dependent FUBI interactors.

**Figure:**
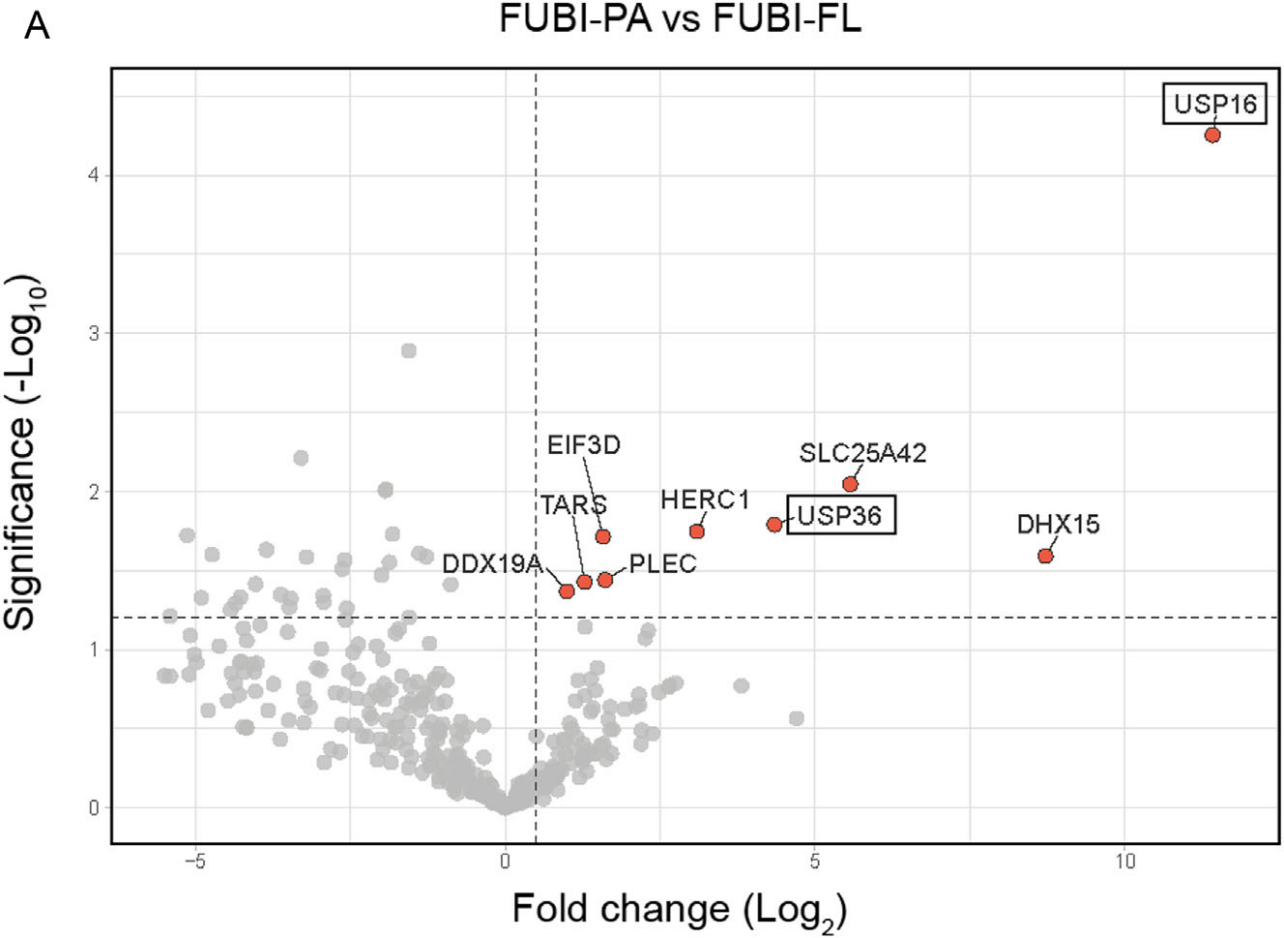

**Figure:**
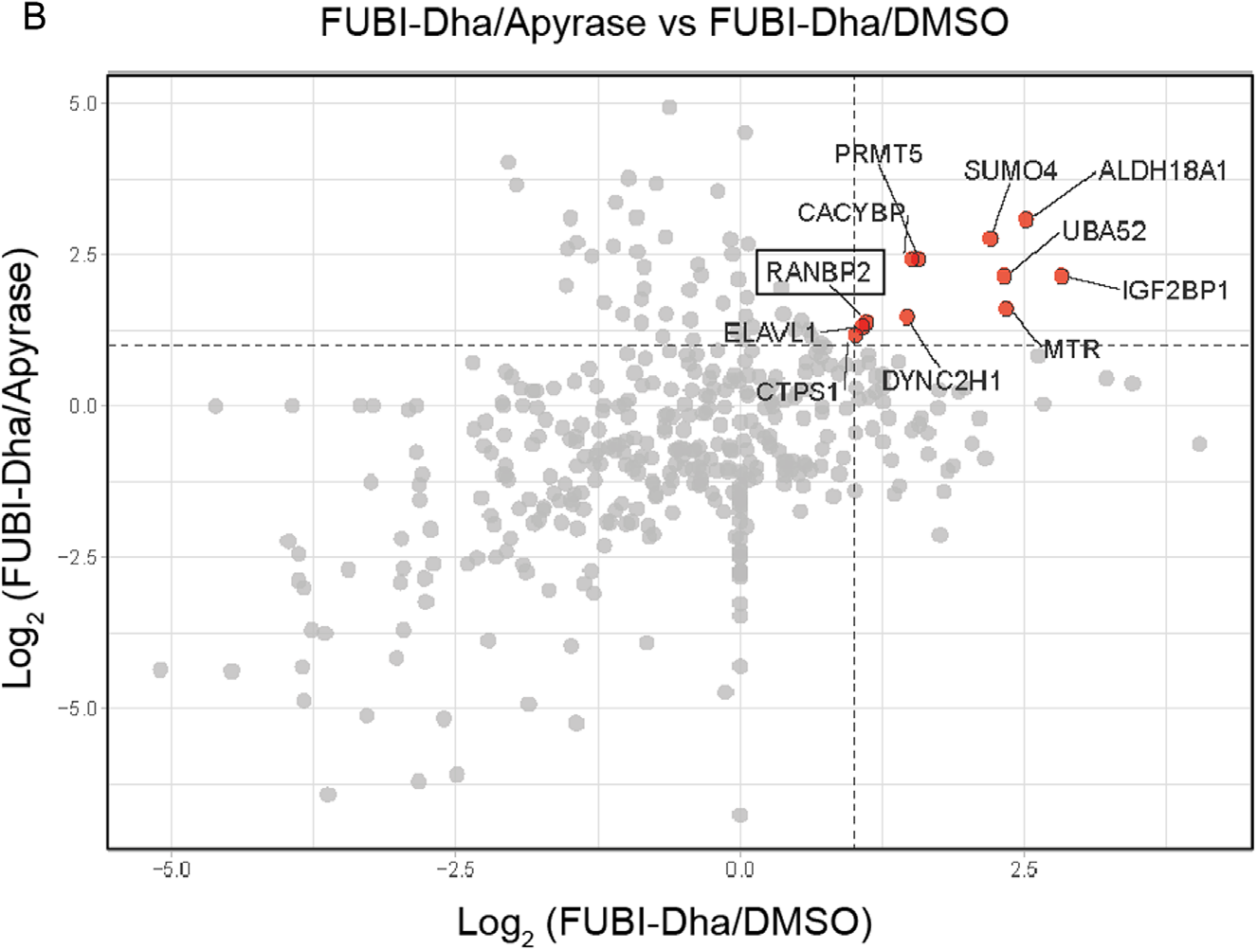

Next, we utilized our synthetic FUBI‐Dha for pulldown assays in HEK293Tlysates to profile FUBI‐related conjugation enzymes (Figure 3B, S5, S6, Supporting Information). To leverage the ATP‐dependent reactivity of our probe, we conducted affinity‐based proteomic profiling (ABPP) both in the presence of ATP and with apyrase‐mediated ATP‐depletion. Proteomics hits were filtered based on their domains and functions associated with conjugation. Except for RANBP2, a known SUMO E3 ligase,^[^ ^31^ ^]^ no other conjugation enzymes were identified, thereby limiting further characterization through in vitro FUBIylation assays, which require a complete set of cascading components (E1, E2, E3) for reconstitution. It is currently unknown whether specific cellular conditions (e.g., interferon stimulation^[^ ^7^ ^]^) are necessary for FUBIylation to take place and our synthetic FUBI‐Dha provides a valuable tool for further investigation.

### 2.2 Toward Profiling FUBI Chain Interactors

**Figure 4:**
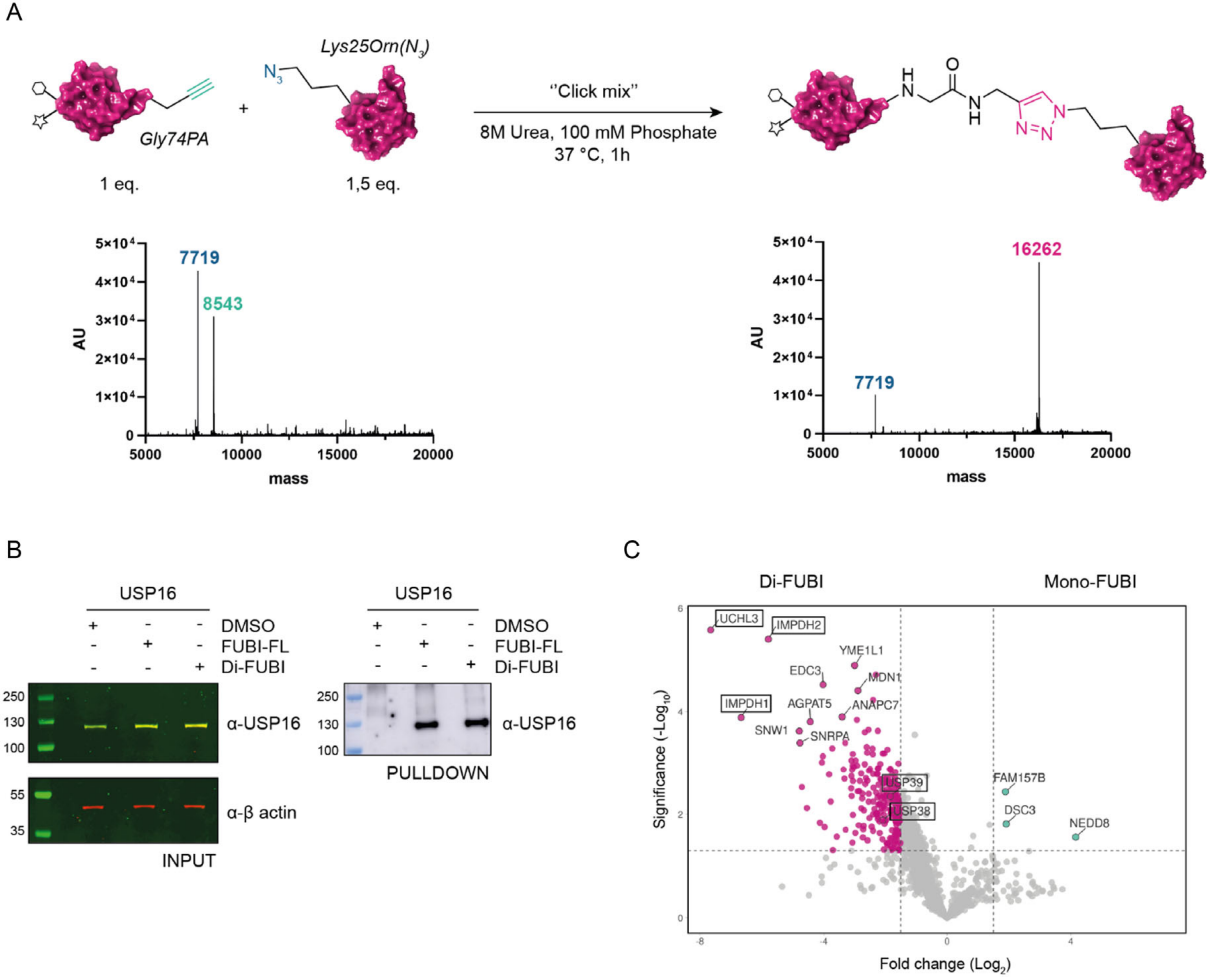
Synthesis and application of Rho/Biotin‐triazole‐linked Di‐FUBI (uncleavable Di‐FUBI). A) Uncleavable Di‐FUBI was synthesized via CuAAC using Rho/Biotin‐FUBI‐PA (1 eq, MW = 8543) and FUBI‐K25Orn(N3) (1.5 eq, MW = 7719), both prepared by SPPS (Figure S7, Supporting Information). The reaction, performed under denaturing conditions with “click mixture” (100 mM CuSO4, 600 mM Sodium Ascorbate, 100 mM TBTA ester), yielded the triazole‐linked product (MW = 16 262), confirmed by LC‐MS analysis. B) Pulldown of ectopically expressed FLAG‐USP16 in HEK293T lysates with FUBI‐FL and uncleavable Di‐FUBI. C) Proteomics analysis from non‐covalent pulldown with uncleavable Di‐FUBI in HEK293T cell lysates. FUBI‐FL (negative control) was used to distinguish Mono‐ and Di‐FUBI‐ interactors (Figure S9, Supporting Information).

With the aim of assessing specific interactors involved in the regulation of FUBI chains, we noted that, similar to Ub, FUBI features the C‐terminal DiGly motif (Figure 1A, S1A, Supporting Information) as a key recognition site for conjugation and deconjugation enzymes in the formation and disruption of the isopeptide bond.^[^ ^3^ ^]^ In contrast to Ub, FUBI contains only one internal Lys at position 25 (Figure S1A, Supporting Information), which serves as the candidate residue for isopeptide‐linked poly‐FUBI chain formation. To profile cellular interactors responsible for FUBI chains modulation, we aimed to generate a Di‐FUBI probe. Therefore, we expanded our toolbox with Rho/Biotin‐K25‐triazole‐linked Di‐FUBI (uncleavable Di‐FUBI) (**Figure** **4**A). The triazole linkage, a stable isostere of the native Gly‐ε‐Lys isopeptide bond, is resistant to proteolytic cleavage by proteases, making it a valuable uncleavable mimic of native Di‐FUBI.^[^ ^32^ ^]^ Triazole‐linked Di‐FUBI (uncleavable Di‐FUBI) was synthesized using copper(I)‐catalyzed azide‐alkyne cycloaddition (CuAAC), employing FUBI‐PA (1 eq) and FUBI‐K25Orn(N_3_) (1.5 eq), which was prepared by linear SPPS (Figure S7, Supporting Information). The reaction was performed at 37 °C under denaturing conditions (8 M urea, 100 mM phosphate) with a “click mixture’’ (100 mM CuSO_4,_ 600 mM Sodium Ascorbate, 100 mM TBTA ester).^[^ ^33^, ^34^ ^]^ After 1 h of incubation, LC‐MS analysis confirmed the complete conversion of Rho/Biotin‐FUBI‐PA into the triazole‐linked Di‐FUBI, which was then purified by HPLC (Figure 4A, S3, Supporting Information).

Subsequently, we evaluated the ability of synthetic triazole‐linked Di‐FUBI to be recognized by the FUBI‐related proteases USP16 and USP36 using pulldown assays in HEK293T lysate. Effective targeting and capturing of ectopically expressed full‐length FLAG‐USP16 and FLAG‐USP36 was visualized by in‐gel fluorescence scanning followed by immunoblotting (Figure 4B, S8, Supporting Information). Our findings demonstrate that synthetic FUBI‐FL and Di‐FUBI peptides adopt biologically relevant conformations, as evidenced by their effective recognition by the FUBI‐related proteases USP16 (Figure 4B) and USP36 (S8, Supporting Information). Upon this, we used uncleavable Di‐FUBI for proteomic analysis in HEK293T cell lysates to investigate specific interactors involved in the regulation of FUBI chains (Figure 4C, S9, Supporting Information). Among the hits, we identified UCHL3, a DUB belonging to the UCH (ubiquitin carboxy‐terminal hydrolase) family, as the most significant Di‐FUBI interactor. UCHL3 is biologically implicated in DNA damage repair,^[^ ^35^, ^36^, ^37^ ^]^ regulation of the interferon response,^[^ ^38^ ^]^ muscular degeneration,^[^ ^39^ ^]^ and neurogenerative disorders.^[^ ^40^, ^41^ ^]^ Its proteolytic activity has been reported toward several substrates, including NEDD8,^[^ ^42^, ^43^ ^]^ ribosomal proteins (L40 and RPS27a)^[^ ^44^, ^45^ ^]^ and K63‐linked tetra‐Ubiquitinated‐SUMO2.^[^ ^46^ ^]^ Furthermore, we searched for other DUBs among the hits and found USP38 and USP39,^[^ ^47^, ^48^ ^]^ which are involved in various cellular processes such as DNA repair,^[^ ^49^ ^]^ cell cycle regulation,^[^ ^50^, ^51^ ^]^ and immune response.^[^ ^52^, ^53^, ^54^, ^55^ ^]^ Next to the DUBs, we also identified IMPDH1 and IMPDH2 as potential Di‐FUBI regulators. These enzymes function as dehydrogenases, catalyzing the conversion of inosine 5'‐phosphate (IMP) to xanthosine 5'‐phosphate (XMP) in the *de novo* synthesis of guanine nucleotides.^[^ ^56^ ^]^ As key regulators of cell proliferation, IMPDH1 and IMPDH2 have important implications in tumor development and progression.^[^ ^57^, ^58^ ^]^

**Figure 5:**
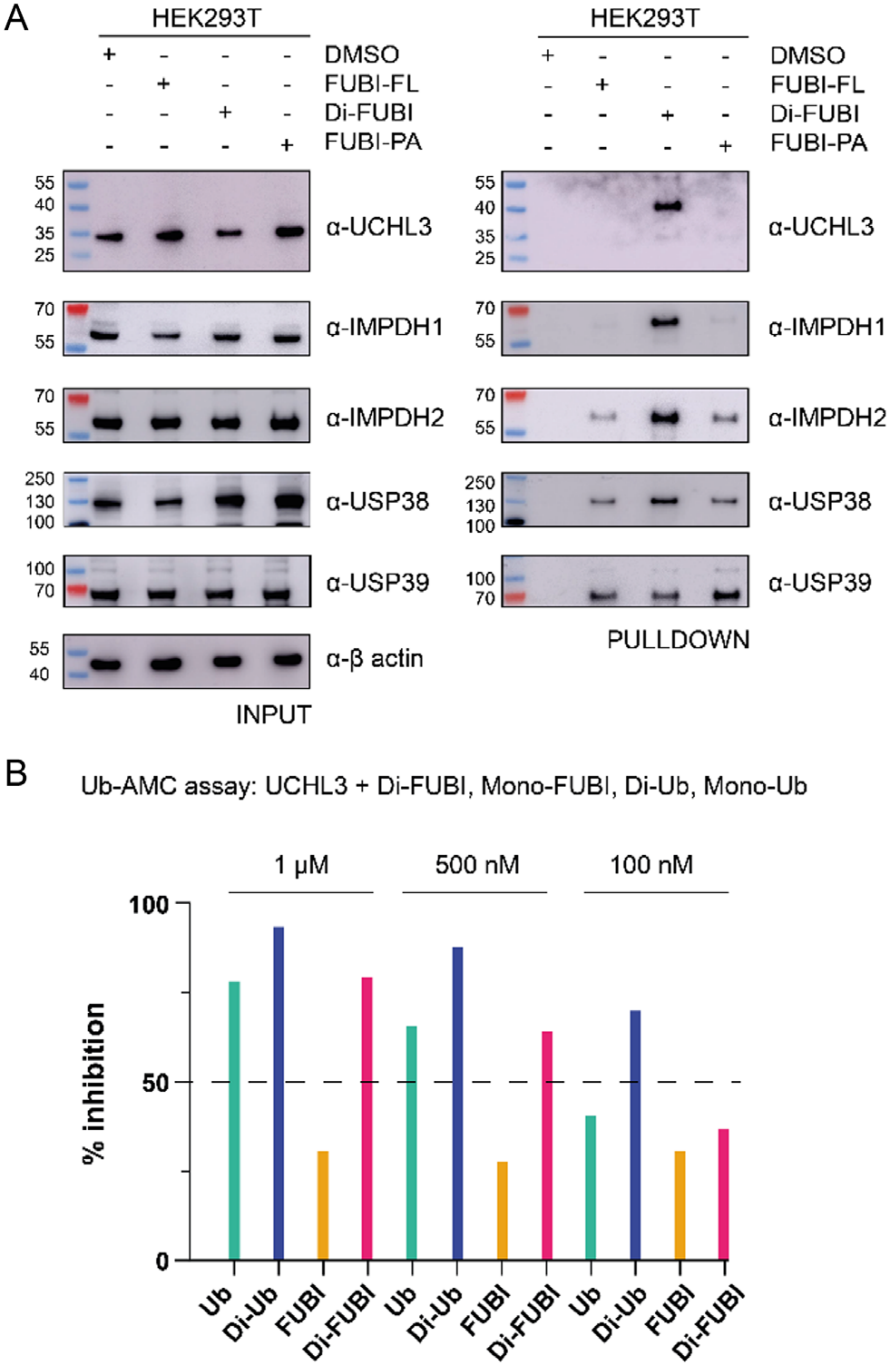
Validation of proteomics hits obtained with uncleavable Di‐FUBI. A) Pulldown of endogenous UCHL3 (Figure S9, Supporting Information), IMPDH1, IMPDH2, USP38, USP39 in HEK293T cell lysate. B) Fluorescence intensity‐based Ub‐AMC cleavage assay showing UCHL3 inhibition in the presence of Ub, Di‐Ub, FUBI, and Di‐FUBI (Figure S11, Supporting Information) at indicated concentrations (t = 30 min).

We then proceeded to validate the newly identified Di‐FUBI interactors (UCHL3, IMPDH1, IMPDH2, USP38, USP39). HEK293T cell lysates were treated with Di‐FUBI, FUBI‐FL, and FUBI‐PA to discriminate between Di‐FUBI/Mono‐FUBI and covalent/non‐covalent interactors, respectively. Pulldown and subsequent western blot analysis revealed IMPDH1 and UCHL3 to be Di‐FUBI specific interactors, whereas USP38, USP39, and IMPDH2 are FUBI interactors (**Figure** **5**A). Notably, UCHL3 has been reported to tightly interact with K27‐linked Di‐Ub, resulting in a conformationally restricted and kinetically trapped bound state which inhibits its enzymatic cleavage activity.^[^ ^59^ ^]^ Given that FUBI's K25 corresponds to Ub's K27 (Figure S1, Supporting Information), we were intrigued to further investigate the interaction of UCHL3 with FUBI. Remarkably, incubation of UCHL3 with K27‐Di‐Ub, followed by SDS‐PAGE and Coomassie staining, previously demonstrated the appearance of a denaturation‐resistant band indicative of a stable inhibitory UCHL3:K27‐Di‐Ub complex.^[^ ^59^ ^]^ In line with this observation, our pulldown data reveals the presence of a shifted UCHL3 band after antibody staining, corresponding in size to the UCHL3:K25‐Di‐FUBI complex (Figure 5A, S10, Supporting Information). To further explore this interaction, we analyzed the effect of Di‐FUBI on the cleavage activity of UCHL3 using a fluorescence intensity‐based assay with Ub‐AMC as the substrate.^[^ ^59^, ^60^ ^]^ Our data show that Di‐FUBI inhibits UCHL3‐mediated cleavage of Ub‐AMC in a concentration‐dependent manner, with potency comparable to Ub (Figure 5B, S11, Supporting Information). These findings suggest a novel regulatory mechanism between UCHL3 and Di‐FUBI.

## 3 Conclusion

In summary, we have developed a robust and scalable linear synthetic platform for producing FUBI‐based peptides (FUBI‐FL, uncleavable Di‐FUBI) and ABPs (FUBI‐PA, FUBI‐Dha), significantly advancing the toolkit available for studying FUBI biology. This platform allows precise incorporation of unnatural building blocks, fluorophores, tags and reactive warheads, providing unparalleled flexibility for tailoring chemical and structural modifications. The adaptability and accessibility of our approach make these FUBI‐chemical tools ideal for facilitating ongoing studies into the poorly understood FUBI machinery.

Our findings suggest a novel regulatory interplay in which Di‐FUBI, mimicking K27‐linked Di‐Ub interactions, forms a stable inhibitory complex with UCHL3, suppressing its enzymatic activity. This points to a potential role for FUBI in influencing deubiquitinating enzyme functions. Pulldown and western blot analyses further identified IMPDH1 and UCHL3 as specific interactors of Di‐FUBI, while USP38, USP39, and IMPDH2 were shown to interact with FUBI. These discoveries provide valuable insights into the regulatory network of FUBIylation.

Although our synthetic platform enables the generation of FUBI‐based peptides and probes, further chemical optimization is needed to overcome challenges such as thioester stability, solubility, and ligation kinetics in synthesizing isopeptide‐linked Di‐FUBI, which is essential for expanding tools to study native Di‐FUBI and poly‐FUBI chains. Nevertheless, the straightforward nature of our experimental setup, combined with the versatility of the dual Rho/Biotin tags, makes these tools readily adoptable for comparative profiling of FUBIylation under various cellular conditions, such as interferon stimulation or other perturbations. Together, this strategy enables a deeper exploration of FUBI biology and holds promise for identifying specific modulators of FUBI‐related enzymes.

This work lays a strong foundation for future research into the mechanisms and functions of FUBIylation. The novel candidates identified through our ABPP experiments present promising opportunities for further investigation, potentially revealing new biological roles and therapeutic targets within the FUBI system.

## Conflict of Interest

The authors declare no conflict of interest

## Supporting information

Supplementary Material

## Data Availability

Data is available in article supplementary material. For any additional information, authors can be contacted. Proteomics data are available via ProteomeXchange with identifier PXD057422.
