# Supplementary material for: Advancing the Exploration of the Ubiquitin‐like Protein FUBI with Synthetic Chemical Tools
Source: Chembiochem. 2025 Jun 23;26(14):e202500321. doi: 10.1002/cbic.202500321 (PMC12278349; doi:10.1002/cbic.202500321)
Supplement: Supplementary file 1 — Supplementary Material [file CBIC-26-e202500321-s001.pdf]

Supporting Information

Advancing the Exploration of the Ubiquitin-like Protein FUBI with Synthetic Chemical Tools

Francesca D'Amico[1], Cami M.P. Talavera Ormeño[1], Shivanganie Poeran[1], Jimmy Akkermans[1], Rayman T.N. Tjokrodirijo[2], Bharath Sampadi [2], Peter Van Veelen[2], Aysegul Sapmaz[1], Monique P.C. Mulder*[1]

[1] Department of Cell and Chemical Biology, Leiden University Medical Center (LUMC), Einthovenweg 20, 2333 ZC Leiden, The Netherlands
[2] Center for Proteomics and Metabolomics, Leiden University Medical Center (LUMC), Albinusdreef 2, 2333 ZA Leiden, The Netherlands
*Corresponding author, email: m.p.c.mulder@lumc.nl

Table of Contents

Figure S1: FUBI and Ub(l) sequences in humans	3
Figure S2: SPPS to obtain Mono-FUBI peptides and probes	4
Figure S3: SDS-PAGE analysis of FUBI peptides and probes synthesized in this work	5
Figure S4: Proteomics results from FUBI-PA pulldown	5
Figure S5: Proteomics experiment with FUBI-Dha	6
Figure S6: Proteomics results from FUBI-Dha pulldown	6
Figure S7: SPPS to obtain uncleavable Di-FUBI	7
Figure S8: Ectopically expressed USP36 pulldown with uncleavable Di-FUBI	7
Figure S9: Proteomics results from uncleavable Di-FUBI pulldown	8
Figure S10: UCHL3 pulldown with uncleavable Di-FUBI (replicates)	8
Figure S11: Ub-AMC FI curves	9
Materials and Methods	10
Chemical Characterization of Peptides and probes	18
Chemical Characterization of Compounds	25
Uncropped gels and western blots	26
References	28

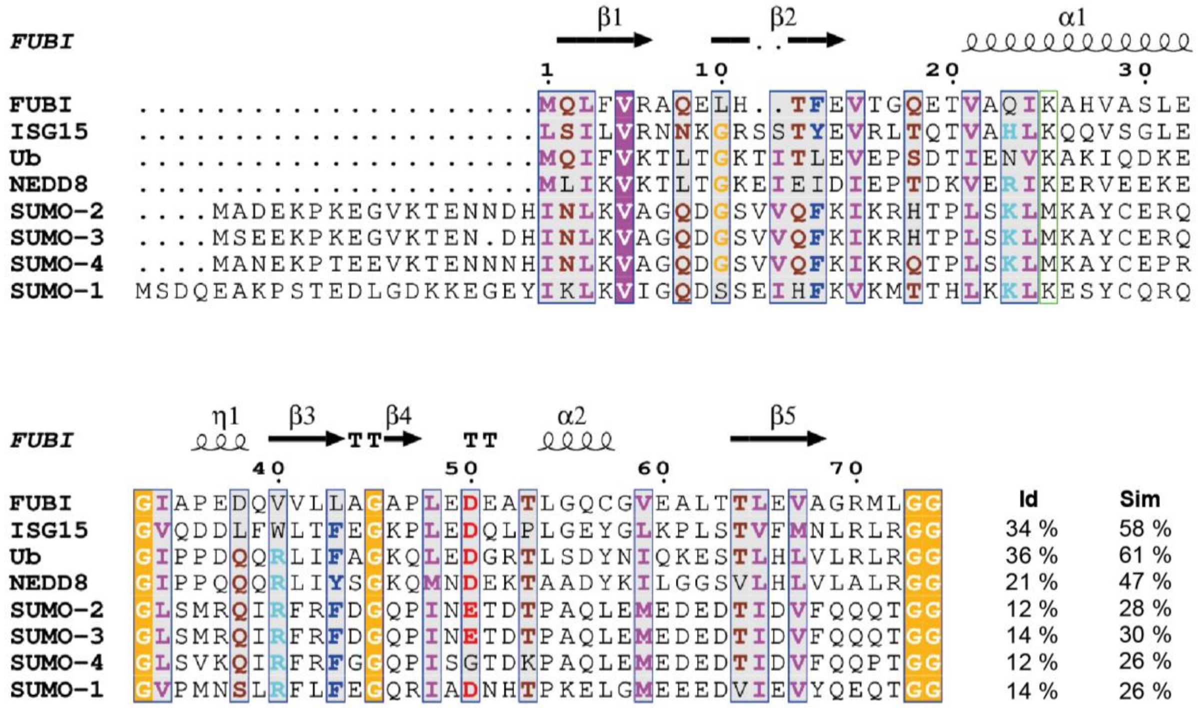

Figure S1. *FUBI and Ub(l) sequences in humans.* All Ubls feature the C-terminal GlyGly motif and contain one or more Lys residues indispensable for the formation (conjugation) and cleavage (deconjugation) of the isopeptide bond. FUBI contains only one internal Lys in position 25 which corresponds to K27 of Ub (green rectangle). Sequence identity (Id) and similarity (Sim) are reported for each protein compared to FUBI.

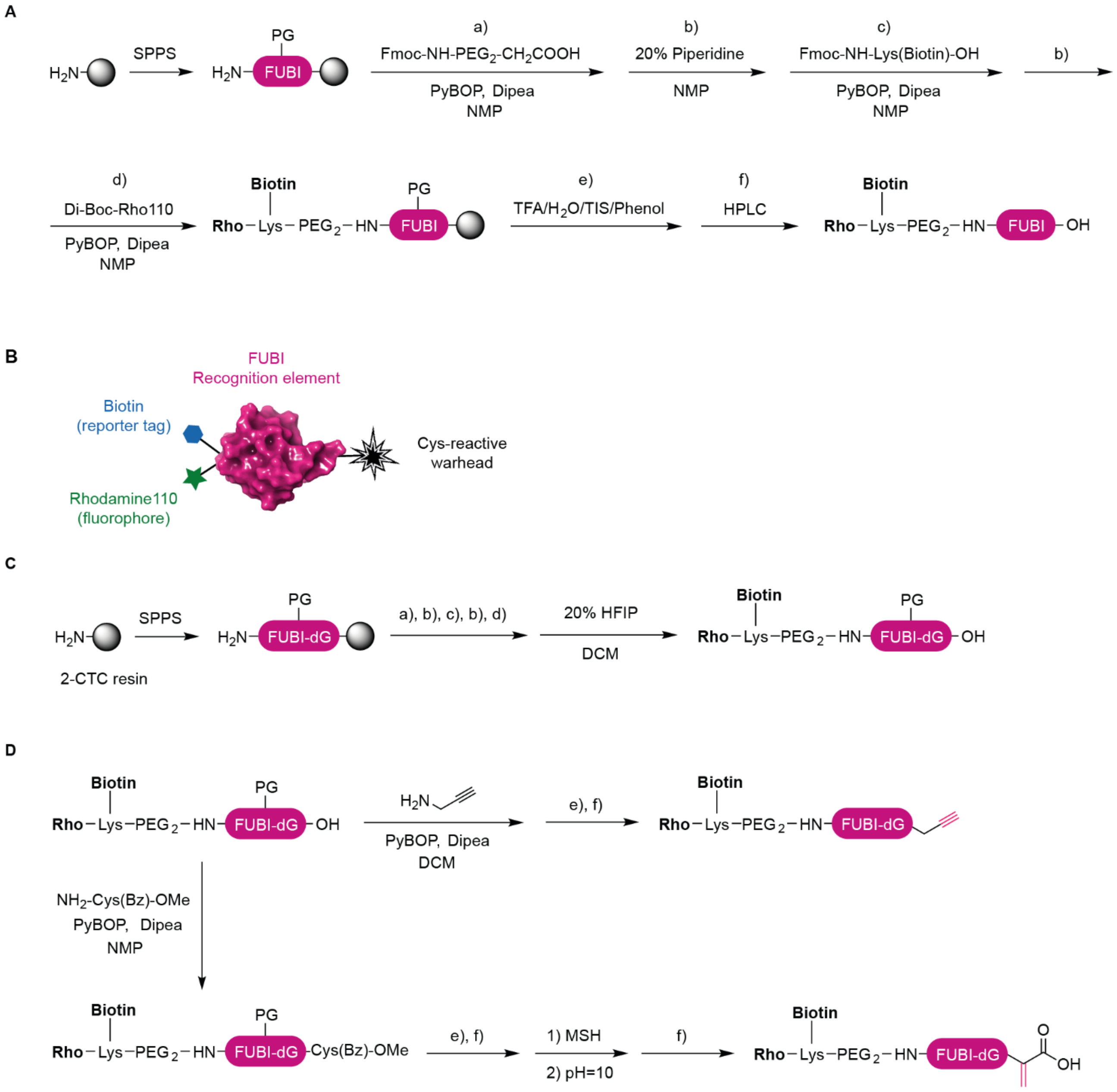

Figure S2: *SPPS to obtain FUBI peptides and probes* **A)** Synthetic scheme to obtain Rho/Biotin-FUBI-FL (LC-MS Analysis Figure S12E). **B)** Design of Mono-FUBI probes synthesized in this work. Synthetic scheme to obtain: **C)** Rho/Biotin-FUBI-PA (LC-MS Analysis Figure S14B) and **D)** Rho-Biotin-FUBI-Dha (LC-MS Analysis Figure S14E).

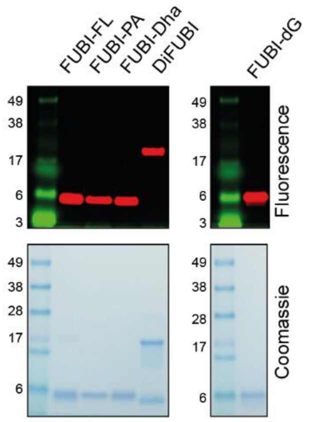

Figure S3: *SDS-PAGE analysis of FUBI peptides and probes synthesized in this work*: Rho/Biotin-FUBI-FL (LC-MS Analysis Figure S12E), Rho/Biotin-FUBI-PA (LC-MS Analysis Figure S14B), Rho/Biotin-FUBI-Dha (LC-MS Analysis Figure S14E), Rho/Biotin-triazole linked Di-FUBI (LC-MS Analysis Figure S15E), Rho/Biotin-FUBI- dG (LC-MS Analysis Figure S13D). *Note:* The residual mono-FUBI present in the purified Di-FUBI sample, arising from a slight excess of the azide-containing FUBI used in the click reaction, does not participate in Neutravidin pulldown experiments nor interferes with fluorescence-based readouts, as it lacks the Rhodamine/Biotin moieties (Fig.S7,Fig.4A).

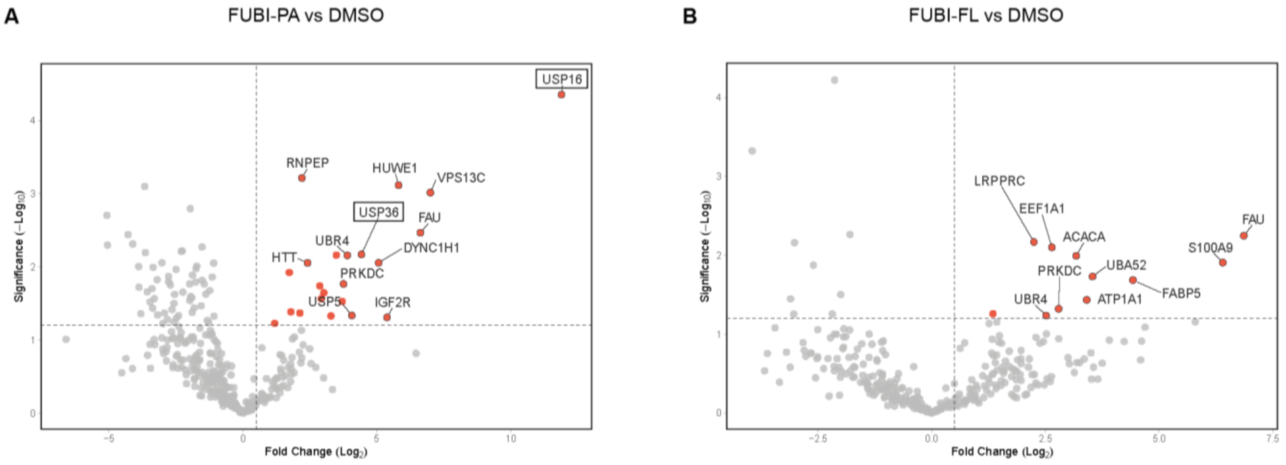

Figure S4: *Proteomics results from FUBI-PA pulldown* (Figure 3A) **A)** with DMSO (negative control 1) used to filter out non-specific interactors and **B)** with FUBI-FL (negative control 2) used to filter out non-covalent interactors.

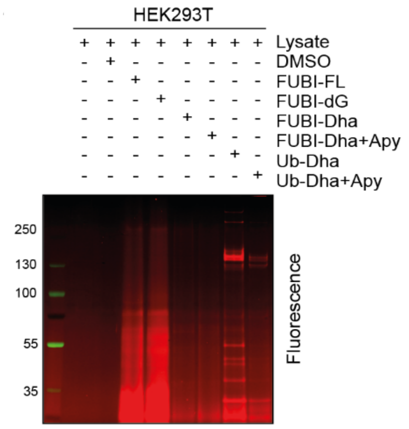

Figure S5: *Proteomics experiment with FUBI-Dha.* Pulldown of endogenous proteins in HEK293T cell lysate (the same samples were submitted for proteomics analysis).

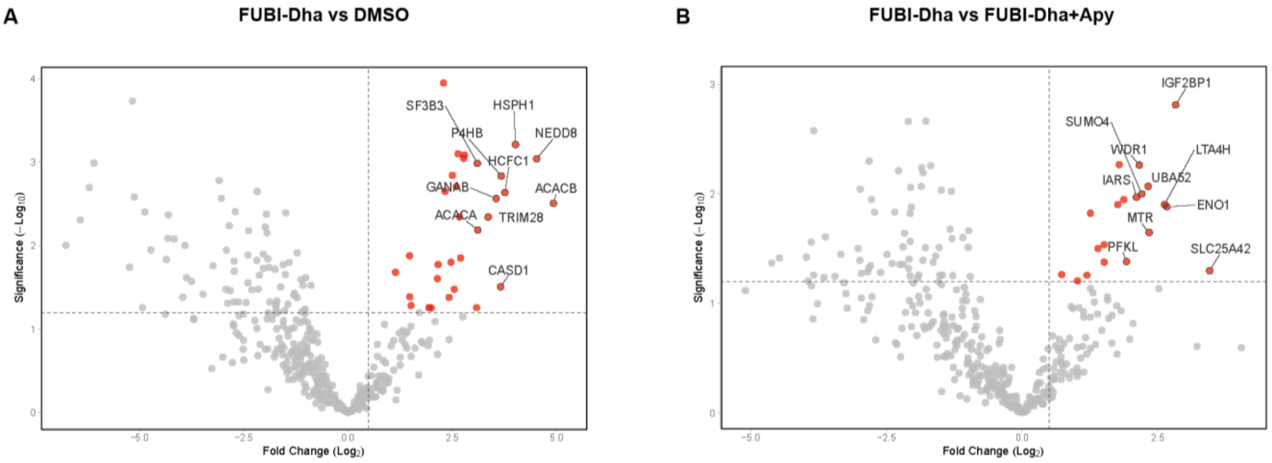

Figure S6: *Proteomics results with FUBI-Dha pulldown* (Figure 3B) **A)** with DMSO (negative control 1) used to filter out non-specific interactors and **B)** with apyrase pre-treatment (negative control 2) used to filter out non-ATP dependent interactors.

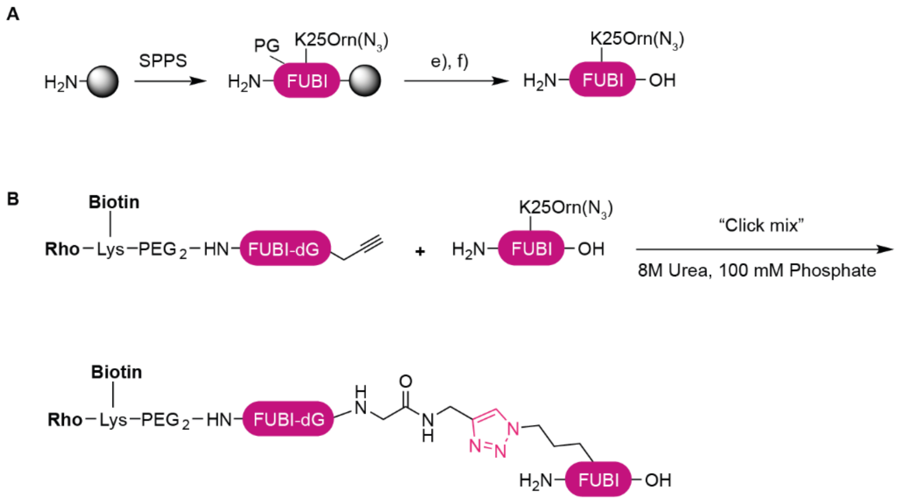

Figure S7: *SPPS to obtain uncleavable Di-FUBI* **A)** Synthetic scheme to obtain FUBI-K25-Orn(N_3) (LC-MS Analysis Figure S15B) and **B)** click reaction to obtain Rho/Biotin-triazole-linked Di-FUBI (LC-MS Analysis Figure S15E). The reaction was performed in denaturing conditions with ''click mixture'' (100 mM $CuSO_4$, 600 mM Sodium Ascorbate, 100 mM TBTA ester) (PG= protecting groups).

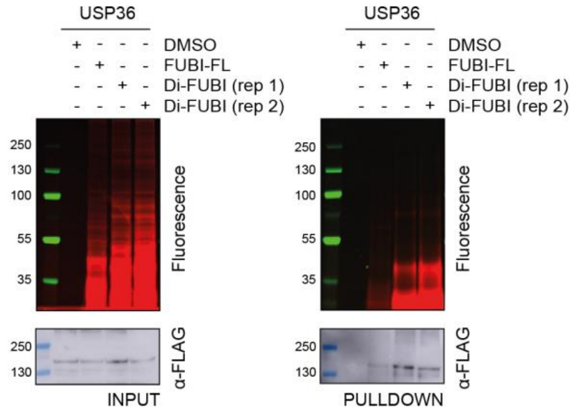

Figure S8: *Ectopically expressed USP36 pulldown with uncleavable Di-FUBI* in HEK293T cell-lysate (Figure 4B).

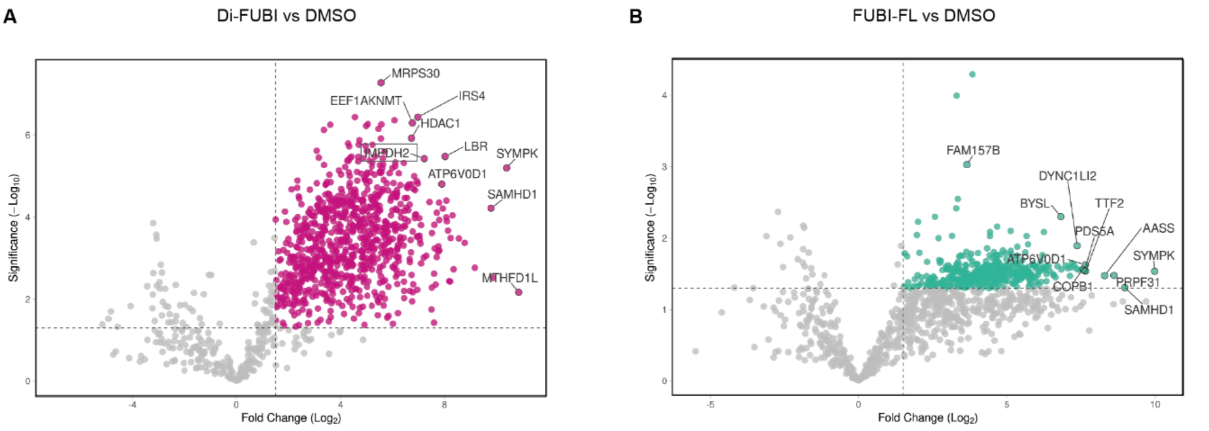

Figure S9: *Proteomics results from uncleavable Di-FUBI pulldown (Figure 4C)* **A)** with DMSO (negative control 1) used to filter out non-specific interactors and **B)** with FUBI-FL (negative control 2) used to discriminate Mono- and Di-FUBI- interactors. The volcano plots were obtained with VolcaNoseR (https://huygens.science.uva.nl/VolcaNoseR/) and the following settings were used as a standard: size of datapoints= 4; Visibility of the data= 0.8; fold change threshold -0.5<x<0.5; significance threshold= 1.3.

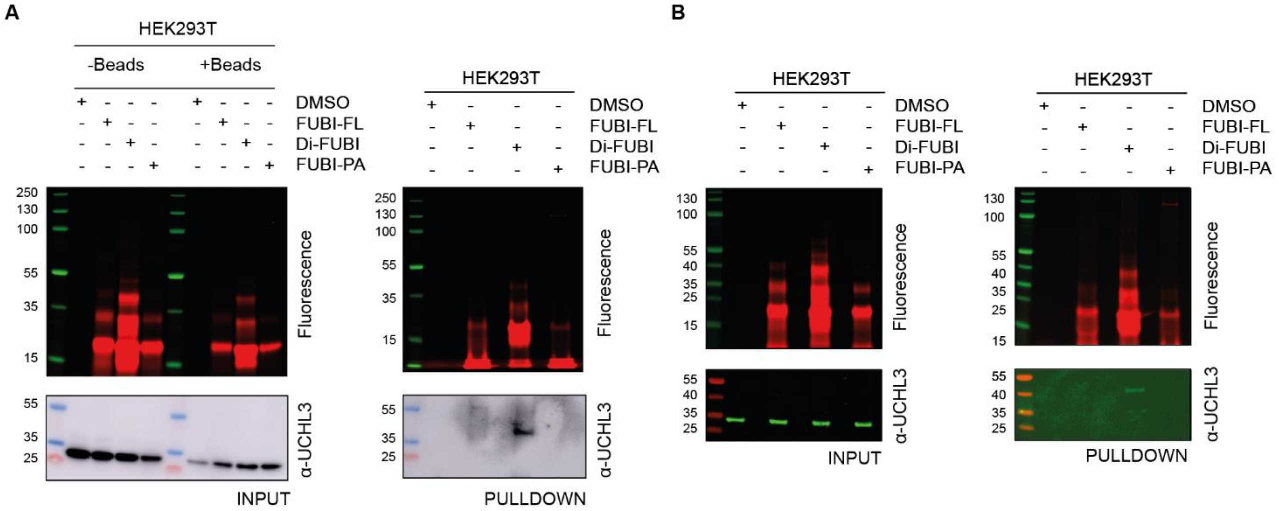

Figure S10: *UCHL3 pulldown with uncleavable Di-FUBI in HEK293T cell lysates* **A)** Biological replicate of Figure 5A **B)** Technical replicate of Figure 5A.

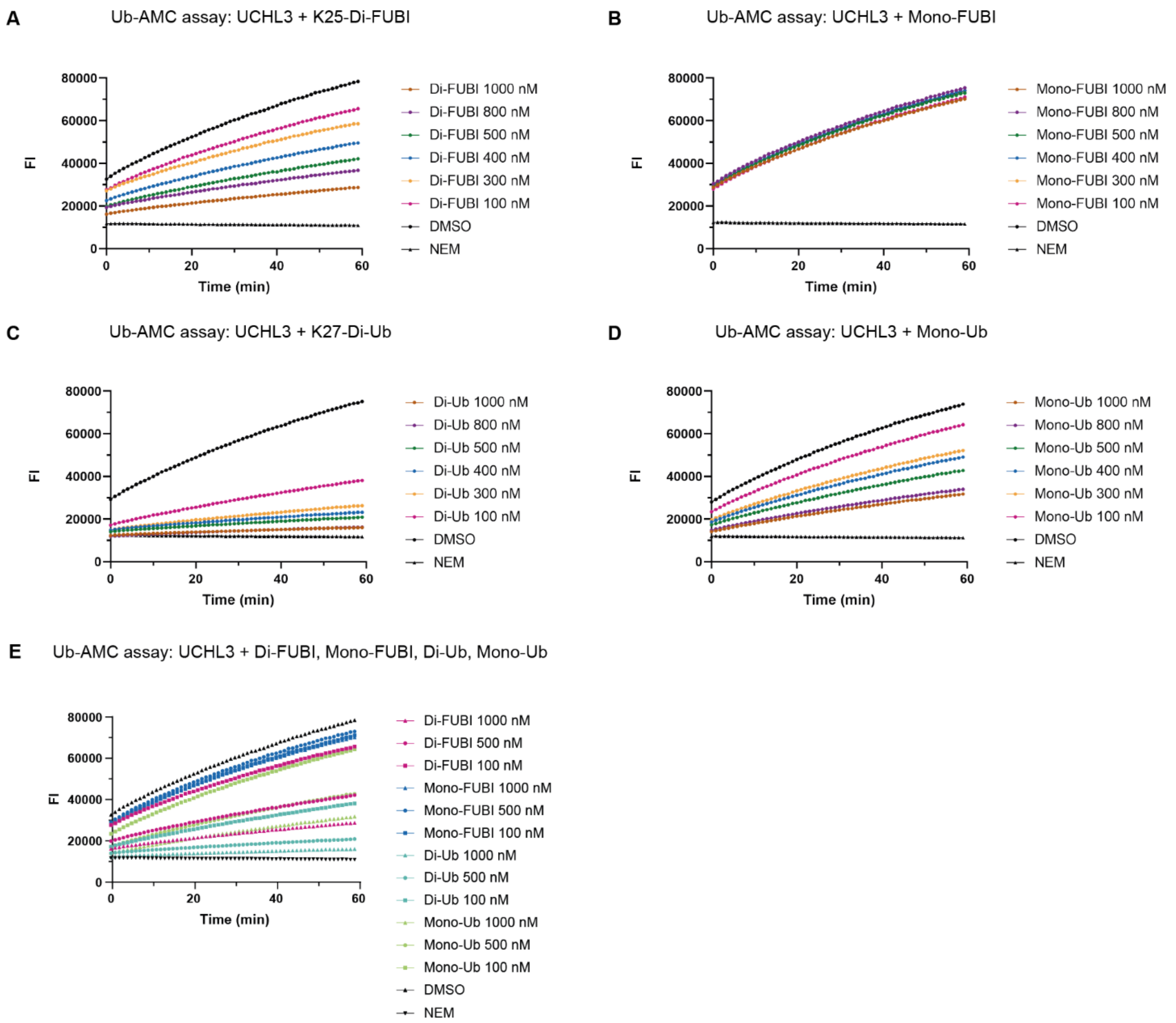

Figure S11: *Fluorescence intensity-based cleavage assay with UCHL3 and Ub-AMC (Figure 5B)* **A)** K25-Di-FUBI titration; **B)** Mono-FUBI titration; **C)** K27-Di-Ub titration **D)** Mono-Ub titration and **E)** Di-FUBI, Mono-FUBI, Di-Ub, Mono-Ub at indicated concentrations. Plots were generated in Graphpad Prism and adapted for illustration using Adobe Illustrator (Adobe Inc.).

Materials and Methods

General procedures

Chemical synthesis

General. Reagents and solvents were obtained from Sigma Aldrich, Combi-Blocks, Iris Biotech and used as received unless stated otherwise.

Solid Phase Peptide Synthesis (SPPS)

Linear SPPS strategy

SPPS was performed on a Syro II MultiSyntech Automated Peptide synthesizer (SYRO robot; MultiSyntech GmbH, Germany) under inert gas (N_2) and vortex application, using standard 9-fluorenylmethoxycarbonyl (Fmoc) based solid phase peptide chemistry on a 20 µmol scale. A four-fold excess of Fmoc-protected amino acid relative to the pre-loaded Fmoc-amino acid TentaGel® TRT resin (0.2 mmol/g, Rapp Polymere, Cat# RA1201, Germany) was used. The resin was washed with Dichloromethane (DCM) (1x 5 ml) and swelled with N-Methyl-2-pyrrolidone (NMP) (1x 1250 µl) for 5 minutes prior to further modifications. Fmoc deprotection was obtained with 20% piperidine in NMP (*v/v*) for 2, 2 and 5 minutes. Fmoc-protected amino acids to-be-coupled (4 eq) were preactivated with PyBOP (4 equiv) and DiPEA (8 equiv) in NMP. Couplings were performed in NMP for 2X25 minutes, followed by Fmoc deprotection with 20% piperidine in NMP for 2×2 and 1×5 min. After each coupling and each removal, resin was washed with NMP (3x 1100 µl). After completion of all coupling/removal cycles the resin was washed with Et_2O, dried under high vacuum and stored for further use.
The methionine (Met) and cysteine (Cys) residues in the sequence were replaced by the known isosteres Norleucine (Nle) and Serine (Ser), respectively.
The following protected pseudoproline and DMB dipeptide building blocks were used during the synthesis of FUBI as indicated in Figure 1A: Fmoc-L-Val-L-Thr($\Psi^{Me,Me}$pro)-OH, Fmoc-L-Ala-(Dmb)Gly-OH, Fmoc-L-Leu-L-Thr(ΨMe,Mepro)-OH.

Note: A persistent truncation of -129 Da, corresponding to loss of Glutamic acid (Glu), was observed during synthesis. Repeated couplings at the E-D-E motif (residues 49-51) with extended coupling times (overnight) were required to achieve full sequence integrity.

General procedure for test cleavage (TC) of peptides

A test cleavage using a small amount of resin was performed to assess the quality of starting materials and the progression of on-resin reactions. The resin was air-dried, treated with ''fast'' test cleavage mix TFA/H_2O/DODT/*i*Pr_3SiH (92.5/2.5/2.5/2.5; *v/v/v/v*; 300 µL) and shaken for 35 min at 37 °C. Samples were filtered and collected in cold Et_2O/*n*-pentane (3/1; *v/v*; 2 mL) to precipitate the product. The precipitate was isolated by centrifugation at 3800 rpm for 2 min at 4°C. The obtained pellet was dissolved in Dimethyl Sulfoxide (DMSO), the DMSO solution was diluted in 0.1% aqueous formic acid and analyzed by LC-MS.

Cleavage from the resin and global deprotection

Peptides were detached from the resin and deprotected as previously described[1]. Briefly, the resin-bound peptides were treated with a freshly prepared solution of TFA/H_2O/*i*Pr_3SiH/Phenol (90:5:2.5:2.5 *v/v/v/v;* 5 ml) for 3 h at rt under gentle shaking, followed by filtration and precipitation in ice-cold Et_2O:*n*-pentane (3/1 *v/v;* 20 ml). After centrifugation at 3800 rpm for 2x15 min at 4 °C, the pellet was dissolved in H_2O:CH_3CN:formic acid (65/25/10; v/v/v; 15 mL) and lyophilized overnight.

Cleavage of protected peptides from the resin

Protected peptides were detached from the resin as previously described[1]. Briefly, the resin was treated with a solution of hexafluoroisopropanol (HFIP) in DCM (1/4; *v/v*; 5 mL) at rt for 2x20 min. The resin was rinsed twice with DCM in between HFIP treatments. Combined filtrates were concentrated

under reduced pressure. Residual HFIP was removed by co-evaporation with DCE (3x12 mL) (to prevent formation of HFIP ester) and the product was lyophilized overnight.

LC-MS analysis

LC-MS measurements were performed on a system equipped with a Waters 2795 Separation Module (Alliance HT), Waters 2996 Photodiode Array Detector (190- 750nm), Phenomenex Kinetex C18 (2.1x50, 2.6 µm) column and LCT™ ESI Mass Spectrometer. Samples were run using 2 mobile phases: A = 1% CH_3CN, 0.1% formic acid in H_2O and B = 1% H_2O, 0.1 % formic acid in CH_3CN, at a flow rate of 0.8 mL/min. Gradient: 0–0.2 min, 2 % B; 0.2–1.8 min, →96 % B; 1.8–2.15 min, 96% B, 2.15–3 min,→2 % B. Data processing was performed using Waters MassLynx Mass Spectrometry Software 4.1 (deconvolution with Maxent1 function).

RP-HPLC purification

The crude peptides were dissolved in a minimal amount of DMSO (max. 10 vol% of the final volume) while heated carefully to increase solubility. The DMSO was added dropwise into H_2O. The pH was checked and adjusted below 7. The mixture was centrifuged (5 min at 3800 rpm). The supernatant was filtered and purified by RP-HPLC. Pure fractions (analyzed by LC-MS) were pooled and lyophilized overnight.

RP-HPLC was performed by using two Systems depending on the material to purify. *System 1* refers to Waters HPLC equipped with a Waters 2489 UV/Vis detector, Waters fraction collector III and Waters XBridge BEH C18 OBD Prep Column (130 Å, 5 µm, 30 × 150 mm). Samples were run at a flowrate of 37.5 mL/min. Mobile phase: A = 1% TFA in H_2O, B = 1% TFA CH_3CN. Fraction collection was triggered by UV intensity (λ = 210 nm).

System 2 refers to Shimadzu LC-20AT HPLC equipped with a Shimadzu SPD-20A UV/Vis detector, a Shimadzu FRC- 10A fraction collector and a Waters XBridge BEH C18 OBD Prep Column (130 Å, 5 µm, 10 × 150 mm). Samples were run at a flowrate of 6.50 mL/min. Mobile phase: A = 0.05% TFA in H_2O and B = 0.05% TFA in CH_3CN, column T = 40 °C.

Synthesis of FUBI

Rho-K(Biotin)-PEG$_2$-FUBI-Full Length

The synthesis of Rho/Biotin-FUBI-FL was performed using the above-mentioned general procedure, sequence and dipeptides. The crude product was purified by preparative HPLC as described in the general procedure (*System 1, 20%→60% B in 18 min*). LC-MS: Rt 1.88 min; ESI MS+ (amu) calcd: 8563, found 8563.3 (deconv.).

Synthesis of FUBI probes

NH_2-FUBI-dG

The synthesis of FUBI-dG was performed following the same procedure described for FUBI-FL by omitting the last Glycine residue. LC-MS: Rt 1.92 min; ESI MS+ (amu) calcd: 7649, found 7649.4 (deconv.).

N-terminal modifications:

Rho-K(Biotin)-PEG$_2$-FUBI-deltaG

The resin-bound FUBI-dG was treated with a solution of Fmoc-NH-PEG$_2$-CH_2COOH (4 eq), PyBOP (4 eq) and DIPEA (8 eq) in NMP and shaken for 3h at rt. After washing with DCM (3x) and NMP (3X), coupling of the spacer was confirmed by TC and LC-MS analysis. Fmoc deprotection was performed as previously described[1]. The resin-bound PEG$_2$-FUBI-dG was treated with a solution of Fmoc-Lys(Biotin)-OH (4 eq), PyBOP (4 eq) and DIPEA (8 eq) in NMP and shaken for 3h at rt. After washing with DCM (3x) and NMP (3X), coupling of the amino acid was confirmed by TC and LC-MS analysis. The resin-bound

K(Biotin)-PEG_2-FUBI-dG was treated with a solution of N,N'-Boc-protected 5-carboxyrhodamine (Rhodamine 110) (4 eq), PyBOP (4 eq) and DIPEA (8 eq) in NMP and shaken for 3h at rt. After washing with DCM (3x) and NMP (3X), coupling of the fluorophore was confirmed by TC and LC-MS analysis. Fmoc deprotection was performed as described above. Cleavage of the protected peptide from the resin was realized as described in the general procedure. LC-MS: Rt 1.96 min; ESI MS+ (amu) calcd: 8506, found 8506 (deconv.).

C-terminal modifications:

Rho-K(Biotin)-PEG_2-FUBI-PA (Prg coupling)

The partially protected Rho-K(Biotin)-PEG_2-FUBI-dG peptide was dissolved in DCM (5 ml) and treated with Propargyl amine (4 eq) in presence of PyBOP (4 eq) and DIPEA (8 eq). The reaction was stirred overnight at rt and the solvent removed *in vacuo;* coupling completion was verified by TC and LC-MS analysis. LC-MS: Rt 1.84 min; ESI MS+ (amu) calcd: 8543, found8543.1 (deconv.). Global deprotection, HPLC purification (*System 1, 30%→65% B in 12 min*) and lyophilization were performed as described in the general procedure.

Rho-K(Biotin)-PEG_2-FUBI-Dha

Rho-K(Biotin)-PEG_2-FUBI-Cys(Bz)OMe (Cys coupling)

The partially protected Rho-K(Biotin)-PEG_2-FUBI-dG peptide was dissolved in DCM (5 ml) and treated with NH_2-Cys(Bz)OMe-OH (4 eq) in presence of PyBOP (4 eq) and DIPEA (8 eq). The reaction was stirred overnight at rt and the solvent removal *in vacuo;* coupling completion was verified by TC and LC-MS analysis. LC-MS: Rt 1.89 min; ESI MS+ (amu) calcd:8714, found 8713.4 (deconv.). Global deprotection, HPLC purification (*System 1, 20%→60% B in 18 min*) and lyophilization were performed as described in the general procedure.

Conversion of Rho-K(Biotin)-PEG_2-FUBI-Cys(Bz)OMe to Rho-K(Biotin)-PEG_2-FUBI-Dha

Conversion of Rho-K(Biotin)-PEG_2-FUBI-Cys(Bz)OMe to Rho-K(Biotin)-PEG_2-FUBI-Dha was performed as previously described[2]. Briefly, purified Rho-K(Biotin)-PEG_2-FUBI-Cys(Bz)-OMe (5 mg) was dissolved in DMSO (200 µl) and diluted in 50 mM phosphate buffer, pH=8 (7.5 ml). A solution of synthesized O-mesitylsulfonyl)hydroxylamine (MSH) in DMF (2.6 mg, 10 eq, 60 mM) was prepared and added to the mixture (150 µL). The reaction was shaken for 1h and monitored by LC-MS. After completion of the amination-elimination mediated by MSH (Rt 1.87 min; ESI MS+ (amu) calcd:8604, found 8604 (deconv.), the pH was increased to 10 and the reaction was shaken overnight at rt to allow ester hydrolysis (Rt 1.89 min; ESI MS+ (amu) calcd:8590, found 8590.2 (deconv.). Next, the reaction was quenched by lowering the pH to 3, diluted in H_2O and purified by RP-HPLC (*System 2, 20%→60% B in 12 min*) as described in the general procedure. LC-MS: Rt 1.87 min; ESI MS+ (amu) calcd: 8590, found 8589.5 (deconv.).

Synthesis of MSH

Ethyl-O-(mesitylsulfonyl)acetohydroxamate (MSH) was synthesized as previously described[3].
Briefly, a solution of ethyl-O-(mesitylsulfonyl)acetohydroxamate (540 mg, 1.89 mmol, 1 eq) in dioxane (0.4 ml) was cooled to 0°C. Perchloric acid ($HClO_4$) (70%, 0.180 ml) was added dropwise over 2 minutes. After stirring for 15 min the solidified white mixture was transferred into ice water (20 ml) and the flask was rinsed with H_2O (5 ml) and Et_2O (5 ml). The clear solution was extracted with Et_2O (1x 10 ml). The organic layer was dried with anhydrous Potassium Carbonate (K_2CO_3) and filtered. The filtrate was then poured into 15 ml of ice-cold petroleum ether and left to crystallize for 1h. The white crystals were isolated by vacuum filtration and transferred to a plastic falcon tube. Lyophilization yielded white needle-like crystals (234 mg, 1,09 mmol, 43%).

^{1}H NMR (300 MHz, CDCl3) δ 2.48 (3H, s, CH3Ar), 2.63 (6H, s, 2 × CH3Ar), 5.72 (2H, br s, NH2), 6.98 (2H, s, Ar-H). ^{13}C NMR (75 MHz, CDCl3): 20.99 (CH3Ar), 22.72 (2 × CH3Ar), 131.28, 131.87, 137.76, 141.16 (Ar). LC-MS calculated for $[C_9H_{13}NO_3S + H]^+$ 216.0616, found 216.0948.

Synthesis of triazole-linked Di-FUBI:

NH_2-FUBI-K25Orn(N_3)

The synthesis of NH_2-FUBI-K25Orn(N_3) was performed by SPPS following the same procedure described for FUBI-FL by replacing the Lys residue in position 25 for Orn(N_3). The crude product was purified by preparative HPLC (*System 1, 60%→90% B in 12 min*) as described in the general procedure. LC-MS: Rt 1.89 min; ESI MS+ (amu) calcd: 7719, found 7719.2 (deconv.).

Rho-K(Biotin)-PEG$_2$-triazole-linked Di-FUBI (Click reaction)

NH_2-FUBI-K25Orn(N_3) (1.5 eq) and Rho-K(Biotin)-PEG$_2$-FUBI-PA (1 eq) were dissolved in DMSO (stock conc. 10 Mm) and added dropwise in denaturing buffer containing 8 M Urea and 100 mM phosphate (starting materials final conc. 0,5 mM and 0,7 mM, respectively). A ''click mix'' was prepared by combining aqueous solutions of Copper sulfate ($CuSO_4$) (100 mM) and Sodium Ascorbate (600 mM), subsequently mixed with Tris(benzyltriazolylmethyl)amine (TBTA) ester (100 mM) in equal volumes, as previously described[4],[5]. The ''click mix'' was added dropwise to the buffer and the reaction was incubated at 37° and shaken for 30 min (550 rpm). After complete conversion to the triazole-linked dipeptide, as verified by LC-MS analysis, the reaction was quenched by addition of 2 M HCl to a final pH of 3 and diluted twice in H_2O to a final concentration of 2 M Urea. The product was purified by HPLC (*System 2, 20%→95% B in 12 min*) and lyophilized as described in the general procedure. LC-MS: Rt 1.82 min; ESI MS+ (amu) calcd: 16263, found 16263 (deconv.).

Procedures for labeling/pulldown experiments

Cell culture

HEK293T (Cat# ATCC® CRL-3216™) and HeLa cell lines were cultured under standard conditions in DMEM (Gibco) supplemented with 10% FCS (Sigma-Aldrich) at 37°C with 5% CO_2. All cell lines have been authenticated and routinely tested for mycoplasma.

Constructs

Human FLAG-HA-USP16 and Flag-HA-USP36 were gifts from Wade Harper[6] (Addgene #22595 and #22579). USP16 C204A and USP36 C131A mutants were generated with the following primers:
USP16: ttcttcaatgcagttatgcagaacttgtc/ ctgcataactgcattgaagaaggctgtgtttcccaaattactgagtc.
USP36: caaccttggcaacaccgcctttctcaatgccac/ ggtgttgccaaggttgtggag).
USP36 was cloned into 2xFLAG-C1 vector (Clontech) using SalI/BamHI restriction sites after amplification with the following primers: caagcttcgaattctgcagtcgacatgccaatagtggataagttgaaggagg and gatcagttatctagatccggtggatccttagcggcgatagctgaggc.

Synthetic peptides and probes

Pure lyophilized peptides and probes were dissolved in DMSO at a stock concentration of 1 mM (FUBI-FL) or 10 mM (FUBI-PA, FUBI-Dha, triazole-linked DiFUBI, Ub-PA, Ub-Dha) and added dropwise to the buffer of choice. The final DMSO concentration was kept as low as possible (1%).

Visualization of Rho/Biotin labeled proteins

Labeled proteins were visualized by in-gel fluorescence using the Typhoon FLA imaging system (GE Healthcare Life Sciences) (λex/λem) 480/530 nm). Gels were stained with Coomassie (Coomassie Instant Blue) and proteins were visualized using a Amersham Imager AI600. Western Blots performed on PVDF membrane were analyzed with Super Signal West Dura Extended Duration Signal Substrate

(ECL, Thermo Fisher) with subsequent visualization using the Amersham Imager AI600; western Blots performed on nitrocellulose membranes were visualized on the LiCOR Odyssey system.

Ectopically expressed FLAG- USP16 and -USP36 pulldown with Rho/Biotin-FUBI-PA

HEK293T cells were transiently transfected with full length FLAG-HA-USP16 (WT or C204A) or 2xFLAG-USP36 (WT or C131A). DNA was delivered into HEK293T cells using polyethylenimine (PEI, Polysciences, Inc.) according to manufacturer's instructions with a transfection time of 24h.
Cells were harvested by scraping with lysis buffer (50 mM TRIS pH 7.5, 150 mM NaCl, 1% NP-40, 5% Glycerol, 2 mM TCEP, 50 U benzonase, 2 mM $MgCl_2$, protease inhibitor tablet (Roche)) and incubated at 4 °C for 1h under gentle shaking. Lysates were clarified by centrifugation (20 min at 4°C, 14000g). Lysates (20 µL) were incubated with the probe (or same DMSO volume) at a final concentration of 10 µM for 1h at 37°C. Reactions were stopped by the addition of 3xSDS-PAGE Loading Buffer (Invitrogen) containing β-mercaptoethanol, followed by boiling for 10 min at 95°C. Samples were resolved using standard SDS-PAGE (4-12% NuPage gel, MOPS buffer) and probe labeling was assessed by in-gel fluorescence scanning using the Typhoon FLA imaging system (GE Healthcare Life Sciences) (λex/λem) 480/530 nm). Samples were transferred onto PVDF membranes and immunoblotting was performed using mouse anti-FLAG (1:1000 dilution; Sigma Aldrich, F3165), or mouse anti-β-actin (1:10000 dilution; Sigma Aldrich, A544) and HRP secondary antibody anti-mouse (1: 5000, Invitrogen, G21040), anti-rabbit (1:5000, Invitrogen, G21234). Results were analyzed with Super Signal West Dura Extended Duration Signal Substrate (ECL, Thermo Fisher) and subsequent visualization using the Amersham Imager AI600.

Endogenous USP16 and USP36 pulldown with Rho/Biotin-FUBI-PA

Cells were harvested by scraping with lysis buffer (50 mM TRIS pH 7.5, 150 mM NaCl, 1% NP-40, 5% Glycerol, 2 mM TCEP, 50 U benzonase, 2 mM $MgCl_2$, protease inhibitor tablet (Roche) and incubated at 4 °C for 1h under gentle shaking. Lysates were clarified by centrifugation (20 min at 4°C, 14000g). Reactions (500 µL) were incubated with the probe (or DMSO) at a final concentration of 10 µM for 1h at 37°C, followed by dilution with lysis buffer to a final volume of 1 ml. High-capacity agarose-neutravidin beads (Thermofisher, 30 µL) were added and the mixture incubated overnight at 4°C under gentle shaking. Beads were washed with: Wash Buffer 1 (2% SDS in H_2O, 2x 8 min, 25 °C), Wash Buffer 2 (50 mM HEPES pH 7.5; 1 mM EDTA; 500 mM NaCl; 1% TRITON-X 100; 0,1% deoxycholate, 1x 8 min, 25 °C); Wash Buffer 3 (10 mM TRIS pH 8.0; 1 mM EDTA; 0,5 % deoxycholate; 0,5 % NP-40; 250 mM LiCl, 1x 8 min, 25 °C); Wash Buffer 4 (50 mM TRIS pH 7.4; 50 mM NaCl , 2x 8 min 25 °C), spun-down for 1 min at 500 g after each washing step. Reactions were stopped by the addition of 3xSDS-PAGE Loading Buffer (Invitrogen) containing β-mercaptoethanol, followed by boiling for 10 min at 95°C. Samples were resolved using standard SDS-PAGE (4-12% NuPage gel, MOPS buffer) and probe labeling was assessed by in-gel fluorescence scanning using the Typhoon FLA imaging system (GE Healthcare Life Sciences) (λex/λem) 480/530 nm). Samples were transferred onto PVDF membranes and immunoblotting was performed using mouse anti-USP16 (1:1000, Bethyl, A301-615A), rabbit anti-USP36 (1:500, Sigma, HPA012082) and HRP secondary antibodies anti-mouse (1:5000, Invitrogen, G21040), anti-rabbit (1:5000, Invitrogen,G21234). Results were analyzed with Super Signal West Dura Extended Duration Signal Substrate (ECL, Thermo Fisher) and subsequent visualization using the Amersham Imager AI600.

Endogenous proteins pulldown with Rho/Biotin-FUBI-Dha

Cells were harvested by scraping with lysis buffer (50 mM HEPES pH 7.5, 100 mM NaCl, 1 mM TCEP, protease inhibitor tablet (Roche) and lysed by sonication. Lysates were clarified by centrifugation (20

min at 4°C, 14000g). Reactions (500 µl) were incubated with the probe (or DMSO) at a final concentration of 10 µM for 1h at 37°C. Indicated samples were incubated with apyrase (2 U) for 15 min at 37°C prior to the addition of the probe (to deplete ATP from the cell lysate and inhibit the conjugation cascade). All samples containing the Dha probe were supplied with ATP and Mg^{2+} every 20 min (first addition 10 mM, subsequent additions 5 mM final concentrations) to favor the conjugation cascade. After dilution with lysis buffer to a final volume of 1 ml, high-capacity agarose-neutravidin beads (Thermofisher, 30 µL) were added and the mixture incubated overnight at 4°C under gentle shaking. Beads were washed with: Wash Buffer 1 (2% SDS in H_2O, 2x 8 min, 25 °C), Wash Buffer 2 (50 mM HEPES pH 7.5; 1 mM EDTA; 500 mM NaCl; 1% TRITON-X 100; 0,1% deoxycholate, 1x 8 min, 25 °C); Wash Buffer 3 (10 mM TRIS pH 8.0; 1 mM EDTA; 0,5 % deoxycholate; 0,5 % NP-40; 250 mM LiCl, 1x 8 min, 25 °C); Wash Buffer 4 (50 mM TRIS pH 7.4; 50 mM NaCl , 2x 8 min 25 °C), spinning-down for 1 min at 500 g and removing the supernatant after each washing step. Reactions were stopped by the addition of 3xSDS-PAGE Loading Buffer (Invitrogen) containing β-mercaptoethanol, followed by boiling for 10 min at 95°C. Samples were resolved using standard SDS-PAGE (4-12% NuPage gel, MOPS buffer) and probe labeling of endogenous proteins was assessed by in-gel fluorescence scanning using the Typhoon FLA imaging system (GE Healthcare Life Sciences) (λex/λem) 480/530 nm).

Ectopically expressed FLAG-USP16 (or endogenous proteins) pulldown with Rho/Biotin-triazole-linked-Di-FUBI

HEK293T cells were transiently transfected with full-length FLAG-USP16 (WT or C204A) where indicated. DNA was delivered into HEK293T cells using polyethylenimine (PEI, Polysciences, Inc.) according to manufacturer's instructions with a transfection time of 24h.

Cells were harvested by scraping with lysis buffer (50 mM TRIS pH=7.5, 150 mM NaCl, 1% NP-40, 5% Glycerol, 2 mM TCEP, 50 U benzonase, 2 mM $MgCl_2$, protease inhibitor tablet (Roche) and incubated at 4 °C for 1h under gentle shaking. Lysates were clarified by centrifugation (20 min at 4°C, 14000 g). Reactions (500 µL) were incubated with the probe (or controls) at a final concentration of 10 µM for 1h at 37°C, followed by dilution with lysis buffer to a final volume of 1 ml. High-capacity agarose-neutravidin beads (Thermofisher, 30 µL) were added and the mixture incubated overnight at 4°C under gentle shaking. Beads were washed four times with lysis buffer (to preserve non-covalent interactions) and spun down for 1 min at 500 g after each washing step. Reactions were stopped by the addition of 3xSDS-PAGE Loading Buffer (Invitrogen) containing β-mercaptoethanol, followed by boiling for 10 min at 95°C. Samples were resolved using standard SDS-PAGE (4-12% NuPage gel) and probe labeling was assessed by in-gel fluorescence scanning using the Typhoon FLA imaging system (GE Healthcare Life Sciences) (λex/λem) 480/530 nm). Samples were transferred onto nitrocellulose membrane (INPUT sample) or PVDF membrane (PULLDOWN sample) and immunoblotting was performed using mouse anti-FLAG (1:1000 dilution; Sigma Aldrich, F3165), mouse anti-USP16 (1:1000, Bethyl, A301-615A), mouse anti-β-actin (1:10000 dilution; Sigma Aldrich, A544) and Fluorescent secondary antibody anti-mouse (1: 5000, Licor, 926-32210) or HRP secondary antibody anti-mouse (1: 5000, Invitrogen, G21040). Results were visualized on a LiCOR Odyssey system (INPUT sample) or with Super Signal West Dura Extended Duration Signal Substrate (ECL, Thermo Fisher) and subsequent visualization using the Amersham Imager AI600 (PULLDOWN sample).

Endogenous proteomics hits cell lysate pulldown (UCHL3, IMPDH1, IMPDH2, USP38 and USP39) with Rho/Biotin-triazole-linked-Di-FUBI

Cells were harvested by scraping with lysis buffer (TRIS pH 7.5 50 mM, NaCl 150 mM, NP-40 1%, Glycerol 5%, TCEP 2 mM, benzonase 50 U, $MgCl_2$ 2 mM, protease inhibitor tablet (Roche) and incubated at 4°C for 1h under gentle shaking. Lysates were clarified by centrifugation (20 min at 4°C, 14000g). Reactions (500 µL) were incubated with the probe (or DMSO) at a final concentration of 10

µM for 1h at 37°C, followed by dilution with lysis buffer to a final volume of 1 ml. High-capacity agarose-neutravidin beads (Thermofisher, 30 µL) were added and the mixture incubated overnight at 4°C under gentle shaking. Beads were washed four times with lysis buffer (not stringent washes to preserve non-covalent interactions) and spun down for 1 min at 500 g after each washing step. Reactions were stopped by the addition of 3xSDS-PAGE Loading Buffer (Invitrogen) containing β-mercaptoethanol, followed by boiling for 10 min at 95°C. Samples were resolved using standard SDS-PAGE (4-12% NuPage gel) and probe labeling was assessed by in-gel fluorescence scanning using the Typhoon FLA imaging system (GE Healthcare Life Sciences) (λex/λem) 480/530 nm). Samples were transferred onto PVDF membranes and immunoblotting was performed using rabbit anti-UCHL3 (1:1000, proteintech, 12384-1-AP), rabbit anti-IMPDH1 (1:1000, proteintech, 22092-1-AP), rabbit anti-IMPDH2 (1:1000, proteintech, 12948-1-AP), rabbit anti-USP38 (1:500, proteintech, 17767-1-AP), rabbit anti-USP39 (1:1000, Bethyl, 23865-1-AP), mouse anti-β-actin (1:10000 dilution; Sigma Aldrich, A544) and HRP secondary antibody anti-mouse (1:5000, Invitrogen, G21040), anti-rabbit (1:5000, Invitrogen, G21234). Results were analyzed with Super Signal West Dura Extended Duration Signal Substrate (ECL, Thermo Fisher) and subsequent visualization using the Amersham Imager AI600.

Ub-AMC fluorescence intensity assay
UCHL3 (10 pM final concentration, 5 µL) was pre-incubated for 30 minutes at room temperature with 5 µL of K25-Di-FUBI, Mono-FUBI, K27-Di-Ub, Mono-Ub (at a final concentration of 1 µM, 800 nM, 500 nM, 400 nM, 300 nM, 100 nM), DMSO (negative inhibition control) or iodoacetamide (positive inhibition control, 10 mM final concentration). Then, Ubiquitin 7-amido-4-methylcoumarin (Ub-AMC) (500 nM final concentration) was added and fluorescence intensity (λex= 350 nm, λem = 450 nm) was measured every 60 seconds for 60 minutes on a Pherastar (BMG Labtech) microplate reader[7]. Each condition was measured in quadruplicate in a 384-well plate using a volume of 15 µL per well. Buffer conditions: 50 mM TRIS pH 7.6, 100 mM NaCl, 2 mM TCEP, 0.05% bovine gamma globulin (BGG), 0.1% 3-[(3-cholamidopropyl)dimethylammonio]-1-propanesulfonate (CHAPS). The data were plotted using GraphPad Prism.

Proteomics experiments

Cell-lysate pulldown with Rho/Biotin-FUBI-PA, Rho/Biotin-FUBI-Dha, Rho/Biotin-triazole-linked-Di-FUBI for proteomics
Labeling and pulldown assays were performed as described above. Gels were briefly run (1,5 cm) and stained with Coomassie (Instant Blue). Proteins were visualized using Amersham Imager AI600.

Processing of protein gel bands for MS analysis
For MS analysis, gel slices were washed, subjected to reduction with dithiothreitol, alkylation with iodoacetamide and in-gel trypsin digestion using a Proteineer DP digestion robot (Bruker). Tryptic peptides were extracted from the gel slices and lyophilized.

Exploris
Peptides were dissolved in water/formic acid (100/0.1 v/v) and analysed by on-line C18 nanoHPLC MS/MS with a system consisting of an Ultimate3000nano gradient HPLC system (Thermo, Bremen, Germany), and an Exploris480 mass spectrometer (Thermo). Samples were injected onto a cartridge precolumn (300 µm × 5 mm, C18 PepMap, 5 um, 100 A, and eluted via a homemade analytical nano-HPLC column (50 cm × 75 µm; Reprosil-Pur C18-AQ 1.9 um, 120 A (Dr. Maisch, Ammerbuch, Germany). The gradient was run from 2% to 40% solvent B (20/80/0.1 water/acetonitrile/formic acid (FA) v/v) in 30 min at 250 nl/min. The nano-HPLC column was drawn to a tip of ∼10 µm and acted as the electrospray needle of the MS source. The mass spectrometer was operated in data-dependent MS/MS mode, with a HCD collision energy at 30% and recording of the MS2 spectrum in the orbitrap,

with a quadrupole isolation width of 1.2 Da. In the master scan (MS1) the resolution was 120,000, the scan range 400-1500, at standard AGC target and a maximum fill time of 50 ms. A lock mass correction on the background ion m/z=445.12003 was used. Precursors were dynamically excluded after n=1 with an exclusion duration of 10 s, and with a precursor range of 20 ppm. Included charge states were 2-5. For MS2 the first mass was set to 110 Da, and the MS2 scan resolution was 30,000 at an AGC target of 100% at a maximum fill time of 60 ms. In a post-analysis process, raw data were first converted to peak lists using Proteome Discoverer version 2.5 (Thermo Scientific), and then submitted to the minimal human Uniprot database (20596 entries), using Mascot v. 2.2.07 (www.matrixscience.com) for protein identification. Mascot searches were done with 10 ppm and 0.02 Da deviation for precursor and fragment mass, respectively, and trypsin was specified as the enzyme. Methionine oxidation and the acetylation (on the protein N-terminus) were set as variable modifications. Carbamidomethyl was set as a fixed modification on cysteines. The false discovery rate was set < 1%. Alternatively, Maxquant version 2.5.1.0 was used with default settings. Data are available via ProteomeXchange with identifier PXD057422.

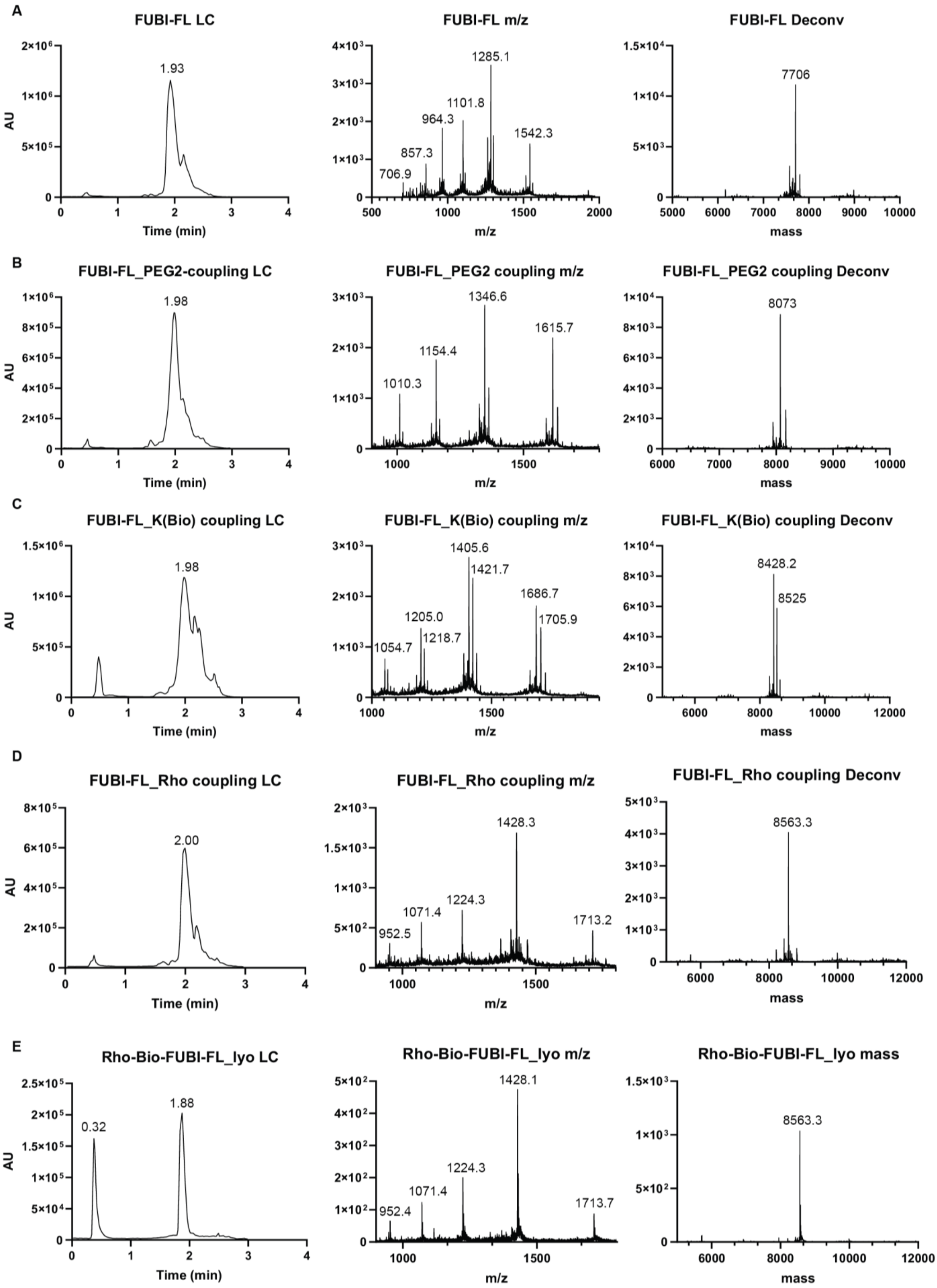
A
FUBI-FL LC
1.93
AU
Time (min)
FUBI-FL m/z
1285.1
1101.8
964.3
857.3
706.9
1542.3
m/z
FUBI-FL Deconv
7706
mass
B
FUBI-FL_PEG2-coupling LC
1.98
AU
Time (min)
FUBI-FL_PEG2 coupling m/z
1346.6
1154.4
1010.3
1615.7
m/z
FUBI-FL_PEG2 coupling Deconv
8073
mass
C
FUBI-FL_K(Bio) coupling LC
1.98
AU
Time (min)
FUBI-FL_K(Bio) coupling m/z
1405.6
1421.7
1205.0
1218.7
1054.7
1686.7
1705.9
m/z
FUBI-FL_K(Bio) coupling Deconv
8428.2
8525
mass
D
FUBI-FL_Rho coupling LC
2.00
AU
Time (min)
FUBI-FL_Rho coupling m/z
1428.3
1224.3
1071.4
952.5
1713.2
m/z
FUBI-FL_Rho coupling Deconv
8563.3
mass
E
Rho-Bio-FUBI-FL_lyo LC
0.32
1.88
AU
Time (min)
Rho-Bio-FUBI-FL_lyo m/z
1428.1
1224.3
1071.4
952.4
1713.7
m/z
Rho-Bio-FUBI-FL_lyo mass
8563.3
mass

Figure S12: (HR)LC-MS analysis of synthesized Rho/Biotin-FUBI-FL. Diode array chromatogram (left; y axis= Absorbance Units), ESI-MS spectrum (middle; y axis= Relative Abundance of the signal intensity to the total ion count) and the deconvoluted mass (right; y axis= Relative Abundance of detected intact molecular species). Figures were generated using Graphpad Prism. **A)** FUBI-FL (TC) **B)** Fmoc-NH-PEG$_2$-CH$_2$COOH coupling (TC) **C)** Fmoc-Lys(Biotin)-OH coupling (TC, the deconvoluted mass +97 corresponds to the TFA-peptide adduct which is removed after purification and lyophilization) **D)** Di-Boc-Rhodamine110 coupling (TC) **E)** Rho/Biotin-FUBI-FL (lyophilized powder after cleavage/global deprotection and HPLC). The LC peak at RT= 0.32 corresponds to DMSO, used as solvent for sample preparation.

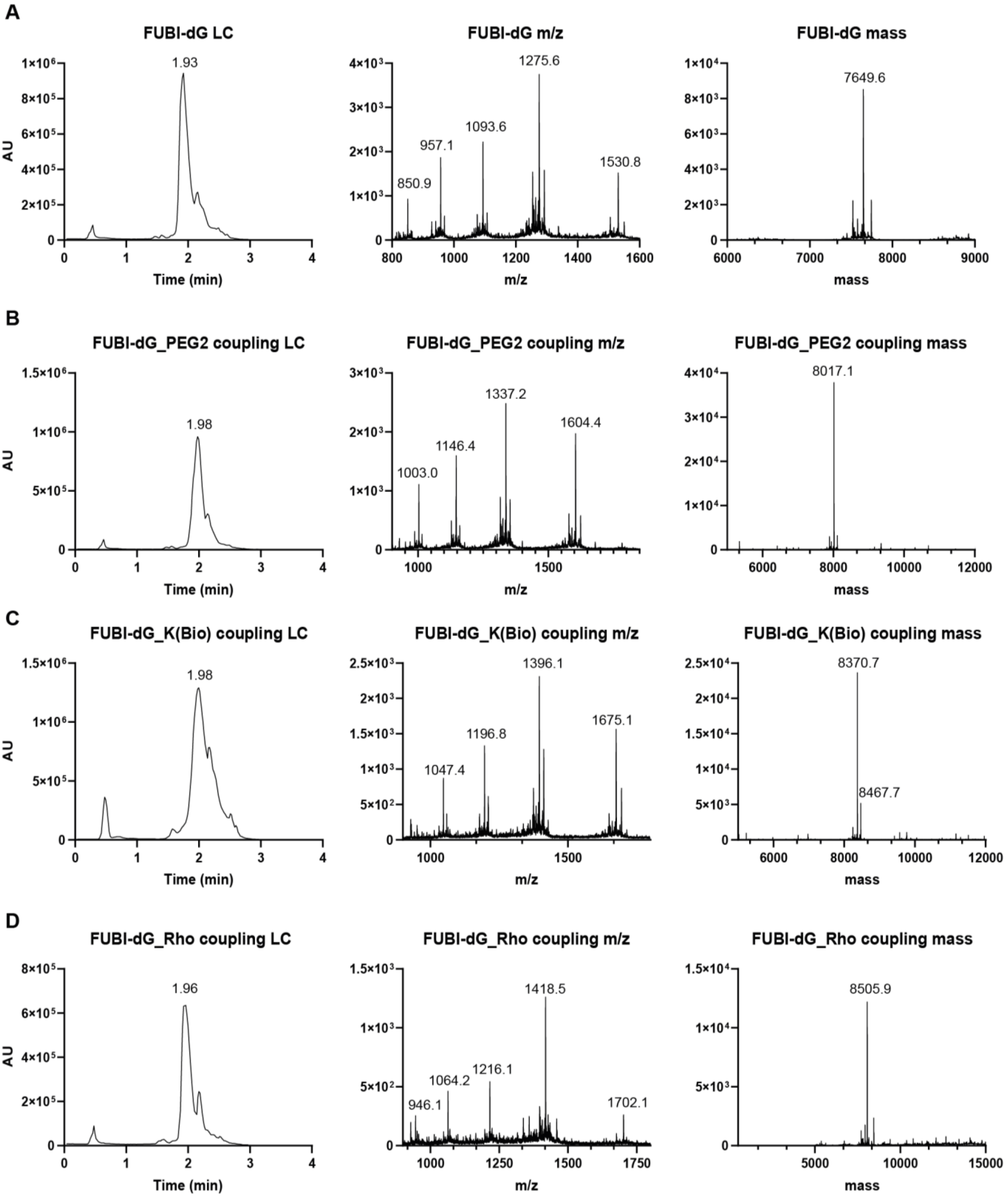

Figure S13: (HR)LC-MS analysis of synthesized Rho/Biotin-FUBI-dG. Diode array chromatogram (left; y axis= Absorbance Units), ESI-MS spectrum (middle; y axis= Relative Abundance of the signal intensity to the total ion count) and the deconvoluted mass (right; y axis= Relative Abundance of detected intact molecular species). Figures were generated by using Graphpad Prism. **A)** FUBI-dG (TC) **B)** Fmoc-NH-PEG_2-CH_2COOH coupling (TC) **C)** Fmoc-Lys(Biotin)-OH coupling (TC) **D)** Di-Boc-Rhodamine110 coupling (TC)**.**

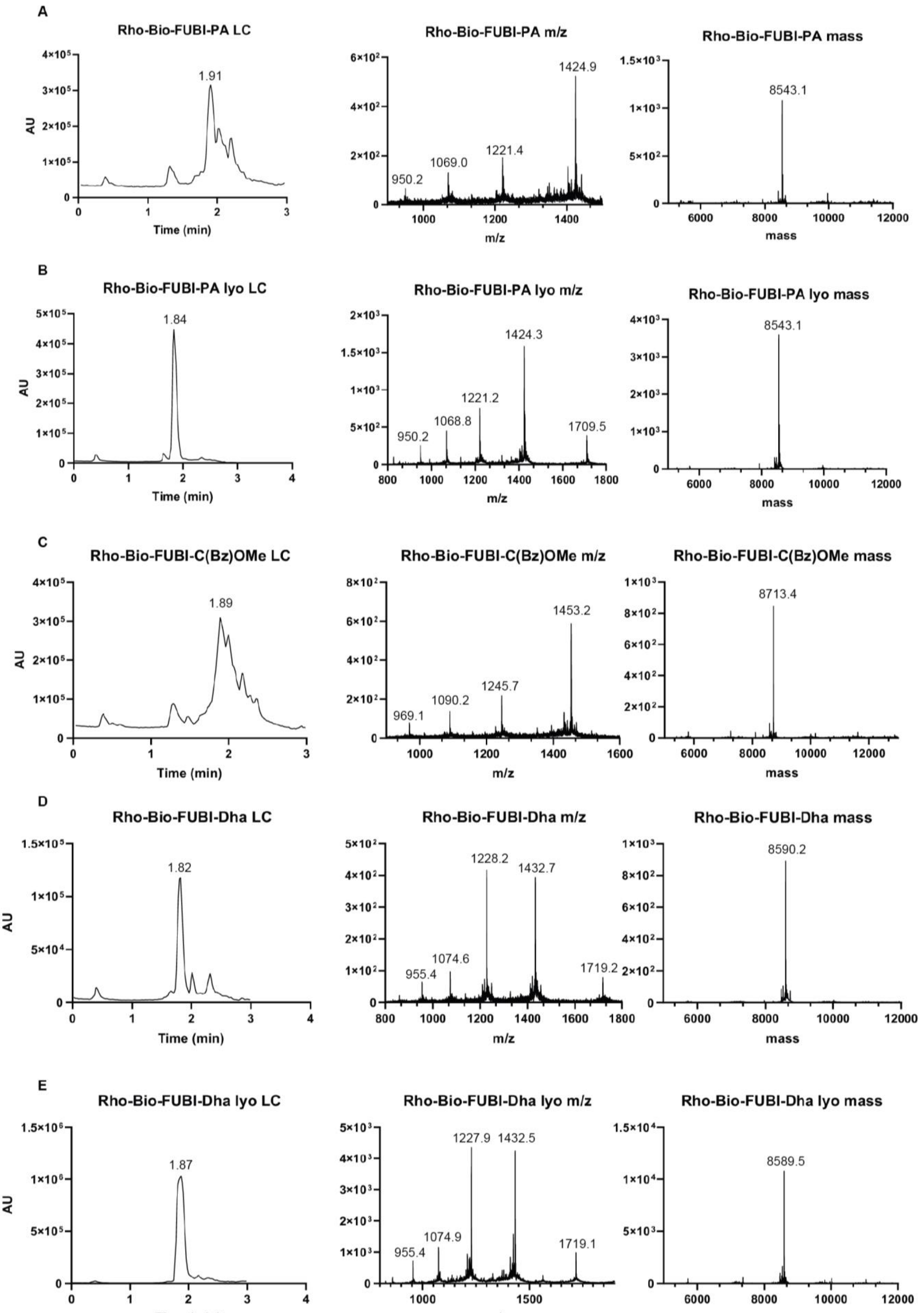
A
Rho-Bio-FUBI-PA LC
1.91
AU
Time (min)
Rho-Bio-FUBI-PA m/z
950.2
1069.0
1221.4
1424.9
m/z
Rho-Bio-FUBI-PA mass
8543.1
mass
B
Rho-Bio-FUBI-PA lyo LC
1.84
AU
Time (min)
Rho-Bio-FUBI-PA lyo m/z
950.2
1068.8
1221.2
1424.3
1709.5
m/z
Rho-Bio-FUBI-PA lyo mass
8543.1
mass
C
Rho-Bio-FUBI-C(Bz)OMe LC
1.89
AU
Time (min)
Rho-Bio-FUBI-C(Bz)OMe m/z
969.1
1090.2
1245.7
1453.2
m/z
Rho-Bio-FUBI-C(Bz)OMe mass
8713.4
mass
D
Rho-Bio-FUBI-Dha LC
1.82
AU
Time (min)
Rho-Bio-FUBI-Dha m/z
955.4
1074.6
1228.2
1432.7
1719.2
m/z
Rho-Bio-FUBI-Dha mass
8590.2
mass
E
Rho-Bio-FUBI-Dha lyo LC
1.87
AU
Time (min)
Rho-Bio-FUBI-Dha lyo m/z
955.4
1074.9
1227.9
1432.5
1719.1
m/z
Rho-Bio-FUBI-Dha lyo mass
8589.5
mass

Figure S14: (HR)LC-MS analysis of C-terminal modifications towards Rho/Biotin-FUBI-PA and Rho/biotin-FUBI-Dha. Diode array chromatogram (left; y axis= Absorbance Units), ESI-MS spectrum (middle; y axis= Relative Abundance of the signal intensity to the total ion count) and the deconvoluted mass (right; y axis= Relative Abundance of detected intact molecular species). Figures were generated by using Graphpad Prism. **A)** Rho/Biotin-FUBI-PA after Propargylamine coupling (TC) **B)** Rho/Biotin-FUBI-PA (lyophilized powder after HPLC). **C)** Rho/Biotin-FUBI-Cys(Bz)OMe after NH_2-Cys(Bz)OMe coupling (TC) **D)** Conversion of Rho/Biotin-FUBI-Cys(Bz)OMe to Rho/Biotin-FUBI-Dha (TC, reaction crude after MSH oxidative amination and ester hydrolysis) **E)** Rho/Biotin-FUBI-Dha (lyophilized powder after HPLC).

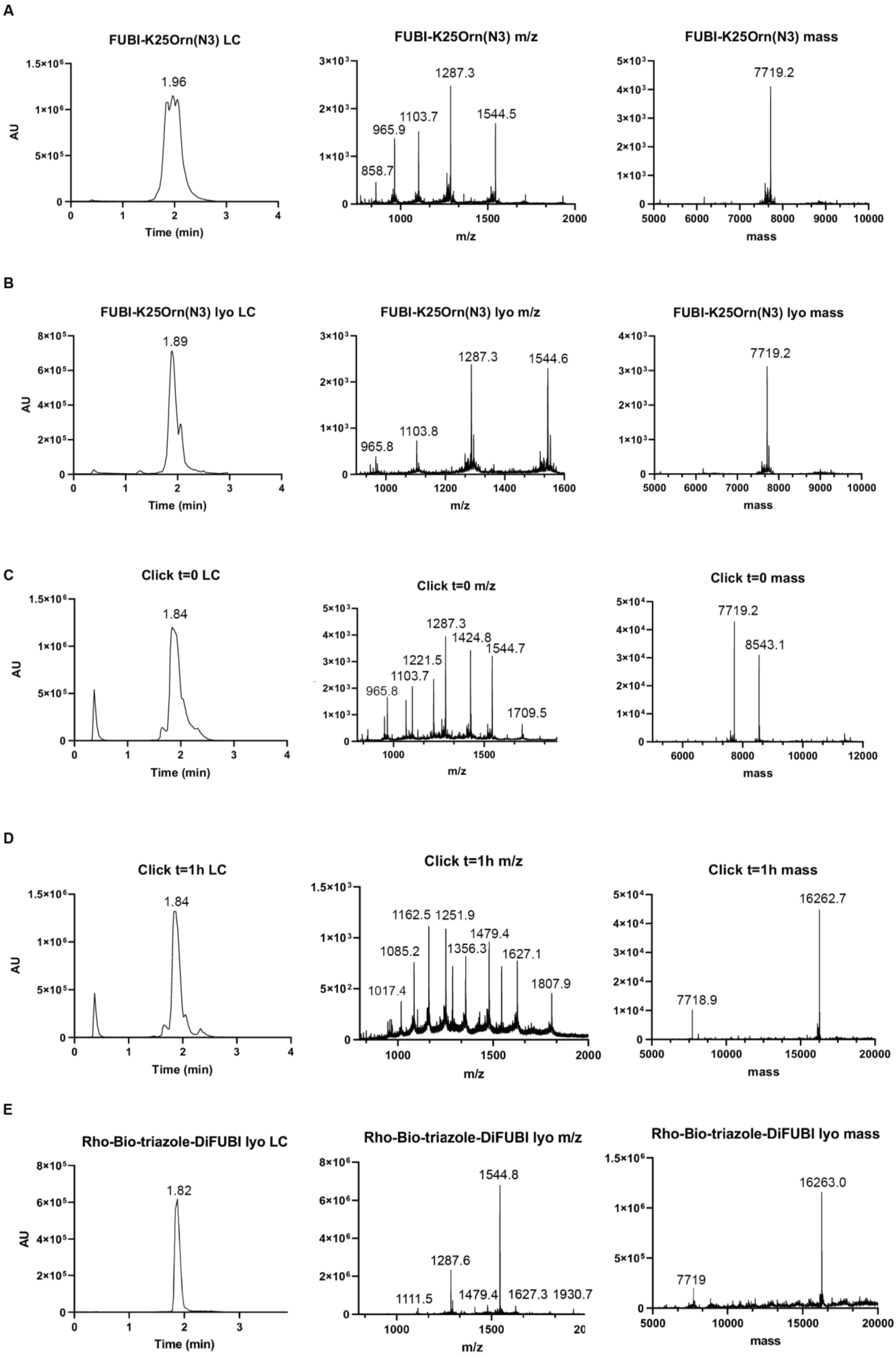
A
FUBI-K25Orn(N3) LC
1.96
AU
Time (min)
FUBI-K25Orn(N3) m/z
1287.3
1103.7
965.9
1544.5
858.7
m/z
FUBI-K25Orn(N3) mass
7719.2
mass
B
FUBI-K25Orn(N3) lyo LC
1.89
AU
Time (min)
FUBI-K25Orn(N3) lyo m/z
1287.3
1544.6
965.8
1103.8
m/z
FUBI-K25Orn(N3) lyo mass
7719.2
mass
C
Click t=0 LC
1.84
AU
Time (min)
Click t=0 m/z
1287.3
1424.8
1544.7
1221.5
1103.7
965.8
1709.5
m/z
Click t=0 mass
7719.2
8543.1
mass
D
Click t=1h LC
1.84
AU
Time (min)
Click t=1h m/z
1162.5
1251.9
1479.4
1085.2
1356.3
1627.1
1017.4
1807.9
m/z
Click t=1h mass
16262.7
7718.9
mass
E
Rho-Bio-triazole-DiFUBI lyo LC
1.82
AU
Time (min)
Rho-Bio-triazole-DiFUBI lyo m/z
1544.8
1287.6
1111.5
1479.4
1627.3
1930.7
m/z
Rho-Bio-triazole-DiFUBI lyo mass
16263.0
7719
mass

Figure S15: (HR)LC-MS analysis of synthesized NH_2-FUBI-K25Orn(N_3) and Rho/Biotin-triazole-linked Di-FUBI. Diode array chromatogram (left; y axis= Absorbance Units), ESI-MS spectrum (middle; y axis= Relative Abundance of the signal intensity to the total ion count) and the deconvoluted mass (right; y axis= Relative Abundance of detected intact molecular species). Figures were generated by using Graphpad Prism. **A)** NH_2-FUBI-K25Orn(N_3) (TC) **B)** NH_2-FUBI-K25Orn(N_3) (lyophilized powder after cleavage/global deprotection and HPLC) **C)** Rho/Biotin-triazole-linked Di-FUBI (TC, click reaction at t=0) **D)** Rho/Biotin-triazole-linked Di-FUBI (TC, click reaction at t=1h) **E)** Rho/Biotin-triazole-linked Di-FUBI (lyophilized powder after HPLC).

Figure S16: ^{1}H-NMR (top) and ^{13}C-NMR (bottom) of synthesized MSH

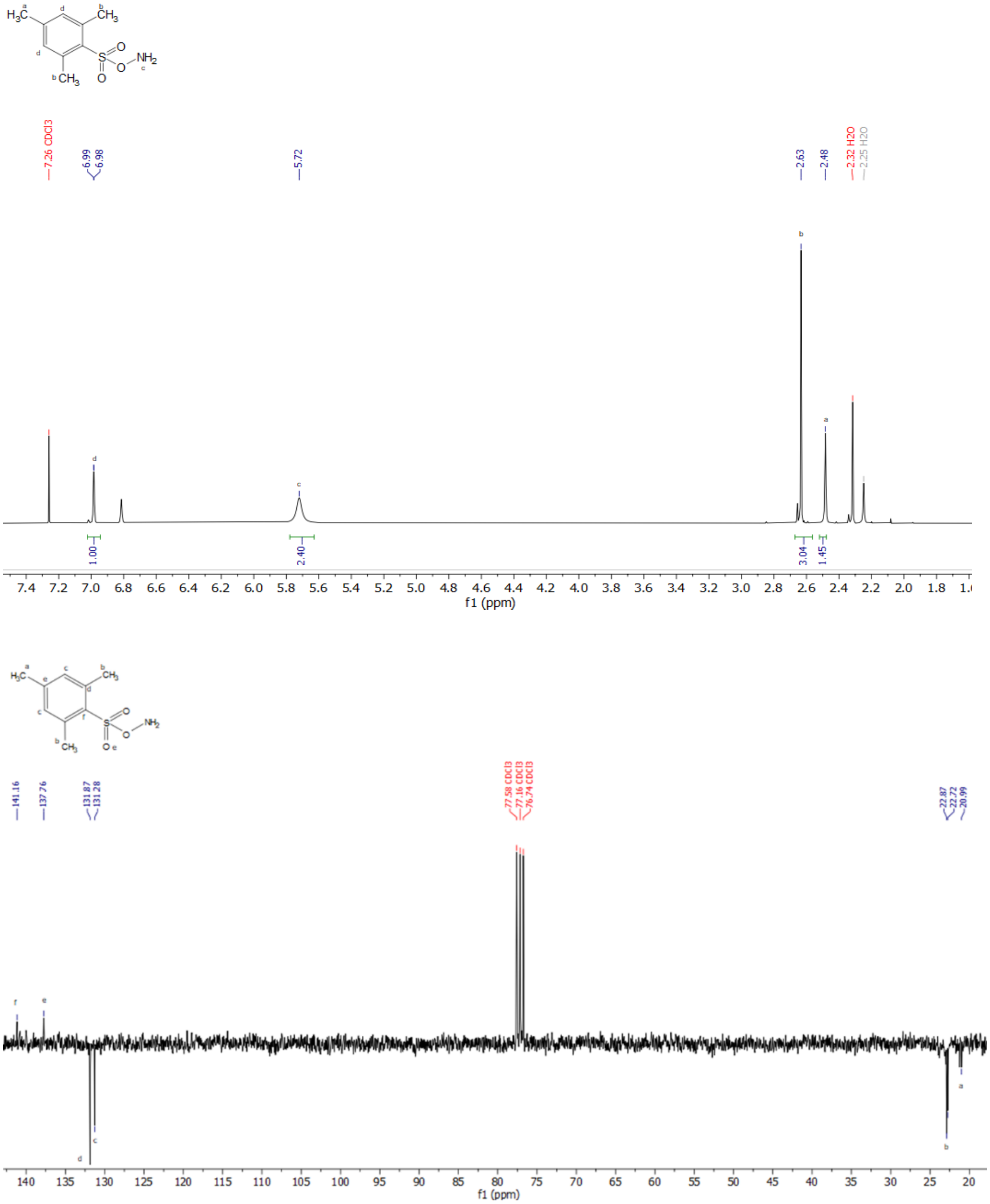

Uncropped gels and western blots

Figure 2A

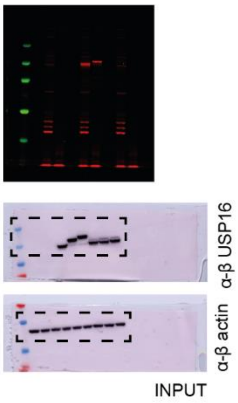

Figure 2B

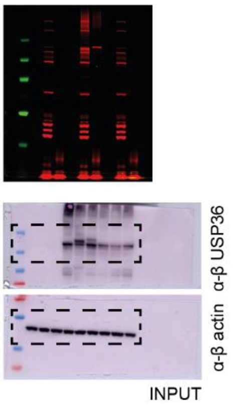

Figure 2C

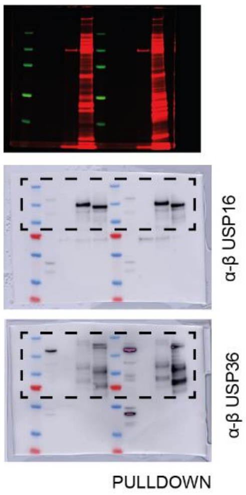

Figure 4B

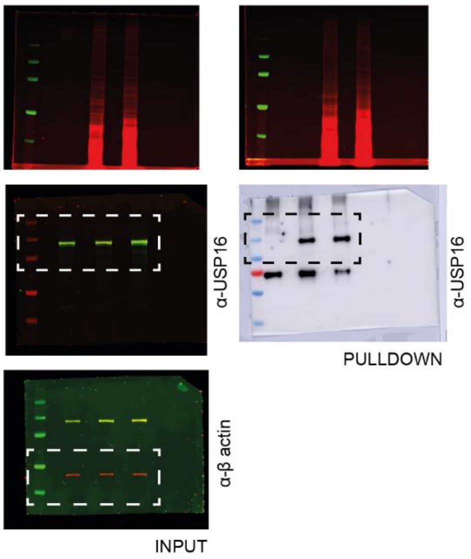

Figure 5A (1)

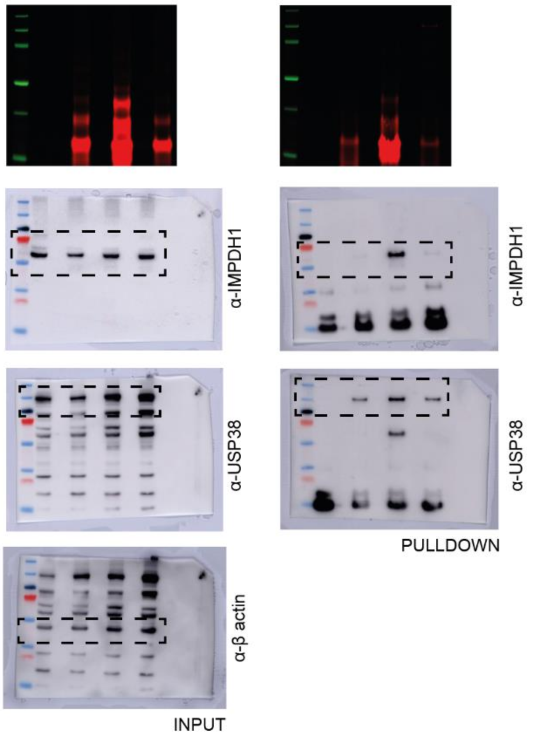

Figure 5A (2)

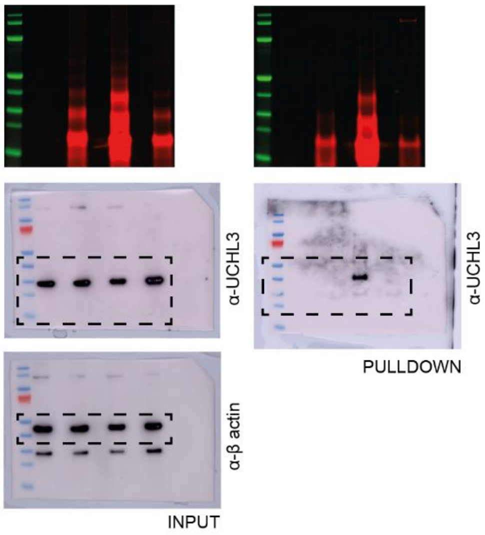

Figure 5A (3)

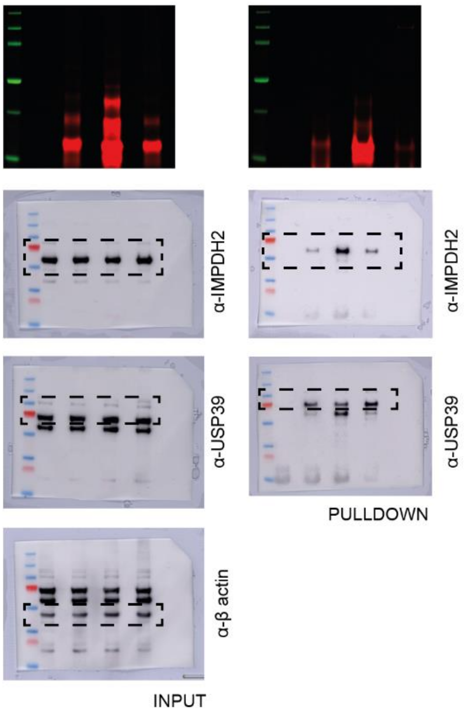

Figure S7

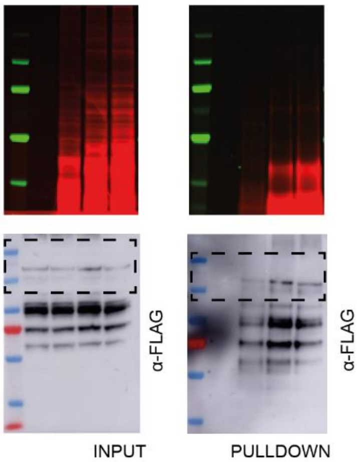

Figure S9A

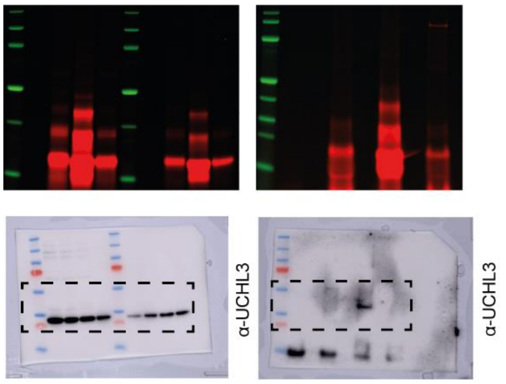

Figure S9B

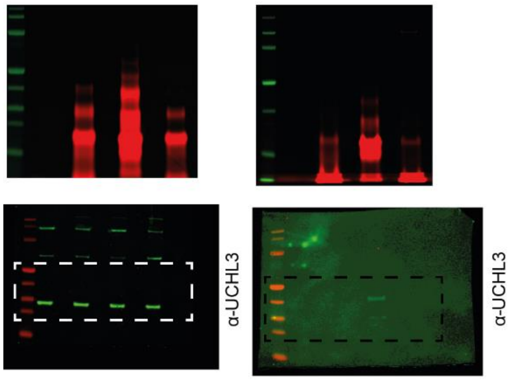

References

[1] F. El Oualid, R. Merkx, R. Ekkebus, D. S. Hameed, J. J. Smit, A. de Jong, H. Hilkmann, T. K. Sixma, H. Ovaa, *Angewandte Chemie International Edition* **2010**, *49*, 10149-10153.

[2] M. P. C. Mulder, K. Witting, I. Berlin, J. N. Pruneda, K.-P. Wu, J.-G. Chang, R. Merkx, J. Bialas, M. Groettrup, A. C. O. Vertegaal, B. A. Schulman, D. Komander, J. Neefjes, F. El Oualid, H. Ovaa, *Nature Chemical Biology* **2016**, *12*, 523-530.

[3] M. Javier, Juan, A., Rincón., Carlos, Mateos., José, Francisco, Soriano., Oscar, de, Frutos., Jeffry, K., Niemeier., Edward, Mark, Davis, **2009**, 263-267.

[4] Z. Zhou, C. J. Fahrni, *Journal of the American Chemical Society* **2004**, *126*, 8862-8863.

[5] D. A. Pérez Berrocal, G. J. van der Heden van Noort, M. P. C. Mulder, *Methods Mol Biol* **2023**, *2602*, 41-49.

[6] M. E. Sowa, E. J. Bennett, S. P. Gygi, J. W. Harper, *Cell* **2009**, *138*, 389-403.

[7] G. B. A. van Tilburg, A. G. Murachelli, A. Fish, G. J. van der Heden van Noort, H. Ovaa, T. K. Sixma, *Cell Chemical Biology* **2021**, *28*, 191-201.e198.
